# Herbivory‐Induced Stomatal Opening Triggers the Dispersal of an Invasive Insect Herbivore

**DOI:** 10.1002/advs.77207

**Published:** 2026-08-17

**Authors:** Limin Chen, Xiaowei Li, Yaru Wang, Farman Ullah, Shuxing Zhou, Lemeng Dong, Weizhong He, Harro J. Bouwmeester, Youming Hou, Ian T. Baldwin, Yaobin Lu

**Affiliations:** ^1^ State Key Laboratory for Quality and Safety of Agro‐products Institute of Plant Protection and Microbiology Key Laboratory of Biotechnology in Plant Protection of the Ministry of Agriculture and Rural Affairs Zhejiang Academy of Agricultural Sciences Hangzhou China; ^2^ Xianghu Laboratory Hangzhou China; ^3^ State Key Laboratory of Agricultural and Forestry Biosecurity Fujian Province Key Laboratory of Insect Ecology College of Plant Protection Key Lab of Biopesticide and Chemical Biology of the Ministry of Education Fujian Agriculture and Forestry University Fuzhou China; ^4^ Lishui Institute of Agriculture and Forestry Sciences Lishui China; ^5^ Plant Hormone Biology Group Swammerdam Institute for Life Sciences University of Amsterdam Amsterdam Netherlands; ^6^ CAS Center for Excellence in Molecular Plant Sciences Shanghai Institute of Plant Physiology and Ecology Chinese Academy of Sciences Shanghai China; ^7^ Department of Molecular Ecology Max Planck Institute for Chemical Ecology Jena Germany

**Keywords:** benzaldehyde, HIPV, *IQM1*, *Phthorimaea absoluta*, stomata

## Abstract

Stomata play important roles in plant responses to phytopathogens, but less is known about their roles in plant‐herbivore interactions. Herbivore damage commonly closes stomata but also elicits the release of herbivory‐induced plant volatiles, important mediators of plant‐herbivore interactions. Here, we examined the responses in tomato plants attacked by the invasive tomato leafminer (*Phthorimaea absoluta*), the larvae of which performed better on infested plants, while ovipositing adults were repelled by larvae‐infested plants, which were distinct from those induced by two other tomato pests, *Bemisia tabaci* and *Spodoptera exigua*. Behavioral assays confirmed that increased releases of volatile benzaldehyde mediated *P. absoluta*’s adult avoidance response, and large‐scale glasshouse trials revealed that the induced release of benzaldehyde was sufficient to trigger long‐distance dispersal of adults. The benzaldehyde release occurred through herbivory‐induced stomatal openings, which were activated by the transcriptional regulation of *IQ‐Motif Containing Protein 1* (*IQM1*). *P. absoluta* population growth and outbreaks were amplified in glasshouse populations of wild‐type plants compared to *IQM1*‐mutated plants. *P. absoluta*’s ability to elicit stomatal opening, as some pathogens do, may have facilitated its rapid range expansion as an invasive pest. Our study illustrates a new pattern in stomata‐herbivore interactions, adding another layer of complexity to stomata‐mediated plant‐herbivore interactions.

## Graphical abstract

The invasive leafminer *Phthorimaea absoluta* uniquely manipulates plant defenses by inducing stomatal opening in tomato. This response, mediated by the stomatal regulator *IQM1*, enhances the release of the repellent volatile benzaldehyde. This volatile triggers the dispersal of adult moths, facilitates colonization of new hosts, and leads to rapid population growth and outbreak formation of *P. absoluta*.

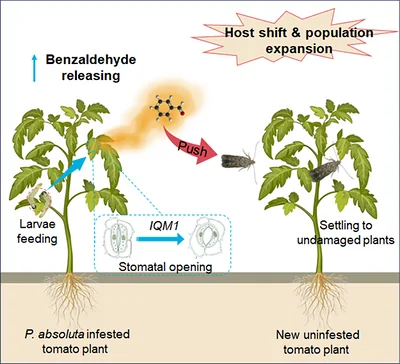

## 1 Introduction

Upon herbivore attack, plants produce and release complex mixtures of volatile organic compounds (VOCs) [1]. These herbivory‐induced plant volatiles (HIPVs) play important roles in interactions between plants and herbivores. HIPVs are known to serve as direct defenses repelling herbivores, and as indirect defenses attracting natural enemies to herbivore‐attacked plants, which benefit both plants and natural enemies [1]. In turn, herbivores can utilize HIPVs for their own benefits [2, 3], for instance, to avoid defended tissues or competition from con‐ or heterospecific herbivores [4, 5, 6]. Alternatively, herbivores that suppress plant defenses can be attracted by HIPVs [7, 8]. Hence, HIPVs play central roles in the co‐evolving arms race between plants and herbivores [9].

While the pathways responsible for HIPV biosynthesis are rapidly being understood, their release mechanisms are less so [1]. The release of plant VOCs, including HIPVs, is controlled by several processes, including production rate [10], their volatility, diffusion across cuticles and membranes [11, 12], and stomatal conductance [13]. HIPV biosynthesis is strongly up‐regulated by herbivory, and increased production triggers HIPV release [14, 15, 16, 17]. Moreover, herbivory‐induced stomatal changes may also be important [18], and stomatal closure is commonly observed in leaves damaged by herbivores with different feeding guilds [19, 20, 21, 22, 23]. Herbivory‐induced stomatal closure has been reported in chewing herbivores *Operophtera brumata* [19] and *Manduca sexta* [24], and sap‐sucking herbivores *Acyrthosiphon pisum* [25] and *Bagrada hilaris* [26]. A recent study of chewing herbivore, *Helicoverpa zea*, larvae feeding on tomato and soybean, revealed a role for salivary glucose oxidase in triggering stomatal closure and inhibiting HIPV emissions [27].

Chewing herbivore damage creates open wounds on plant tissues and causes increased transpiration and water loss [28], while sap‐sucking herbivore feeding decreases the water status of vascular tissues [25], which can both cause water limitation and trigger stomatal closure [29]. In contrast, insects that feed internally, such as leaf miners and gall inducers, can manipulate stomatal development and function in diverse and targeted ways. For example, the leaf‐mining moth *Phyllonorycter blancardella* triggers stomatal closure to increase water‐use efficiency [30], while the leaf‐galling phylloxera *Daktulosphaira vitifoliae* induces the formation of functional stomata on the adaxial leaf surface to promote carbon gain [31]. While stomatal closure under herbivory is documented, whether herbivores can actively induce stomatal opening to manipulate HIPV release remains unknown. To date, no studies have reported herbivory‐induced stomatal opening and HIPV release, and this stands in contrast to the large literature on plant pathogens.

Phytopathogens and their pathogen‐associated molecular patterns (PAMPs) have evolved various strategies to manipulate stomatal apertures [32, 33, 34]. PAMP‐triggered stomatal closure is a commonly observed defense strategy, dubbed “stomatal immunity” or “stomatal defense” [35]. In turn, successful pathogens, including bacterial and fungal pathogens, counter these responses with phytotoxins that reopen stomata and enable pathogens to gain access to mesophyll tissues without damage [36, 37, 38]. Furthermore, pathogen‐produced effectors have also been reported to suppress stomatal immunity by inhibiting stomatal closure or actively opening stomata [35]. Recent studies with the bacterial pathogen *Pseudomonas syringae* reported a close‐open‐close pattern in stomata‐pathogen interaction dynamics, in which, following infection after stomatal re‐opening, pathogen effectors induce abscisic acid (ABA) responses to again close stomata, reducing transpiration in infected leaves and promoting pathogen growth [39, 40].

The tomato leafminer, *Phthorimaea absoluta*, is a devastating invasive pest of tomato crops worldwide [41]. The larvae mine leaves, stems, and fruits, causing complete crop losses depending on the infestation level [41]. Originating in South America, this pest invaded Europe in 2006, spreading rapidly throughout the Afro‐Eurasian supercontinent [42], and now threatens the tomato industry in more than 90 countries [43]. While it feeds on most Solanaceous crops, including potato, eggplant, and tobacco [44], it has a strong preference for tomato [45]. During its rapid range expansion, its populations appear to have escaped controls from higher trophic levels: predators and parasitoids are rarely reported in newly invaded areas [46]. Hence, understanding the interactions between *P. absoluta* and tomato plants may help to understand its rapid range expansion.

Here, we show that *P. absoluta* larvae performed better, while female moths are repelled by larvae‐infested tomato plants. Increased releases of volatile benzaldehyde mediate the adult avoidance response, and large‐scale glasshouse trials reveal this HIPV to be sufficient to trigger long‐distance dispersal. The benzaldehyde release is triggered by herbivory‐induced stomatal openings, which were activated by the transcriptional regulation of *IQ‐Motif Containing Protein 1* (*IQM1*). These do not occur when tomato plants are attacked by two other tomato pests. This work suggests that this herbivore's ability to elicit stomatal opening, much as some pathogens do, may have facilitated its rapid range‐expansion as an invasive pest.

## 2 Results

### 2.1 *P. absoluta* Infestation Increases Conspecific Larval Performance, Adult Dispersal, and Elicits the Release of Specific Hostplant Volatiles

To understand how infestations of *P. absoluta* larvae influence survival, development, and behavior of conspecifics, we compared larval performance on uninfested and larvae‐infested tomato plants. The survival curve (Log‐Rank test: *χ* ^2^ = 4.933, *df* = 1, *p* = 0.0264) (Figure 1A) and pupation rate (*t‐*test: *t* = −2.738, *df* = 40, *p* = 0.009) (Figure S1A) were significantly higher on *P. absoluta* larvae‐infested tomato plants than on uninfested plants, suggesting that larval performance on already infested plants may be an important reason for the *P. absoluta*’s strong preference for tomato. We also examined how infestation affects the oviposition preferences of *P. absoluta* females, as egg‐laying behavior is critical for population establishment and growth. When females were given a choice between uninfested and infested plants, they laid significantly fewer eggs on the infested plants (General Linear Mixed Model (GLMM) test: *χ^2^* = 37.366, *df* = 1, *p* < 0.0001) (Figure S1B).

**FIGURE 1:**
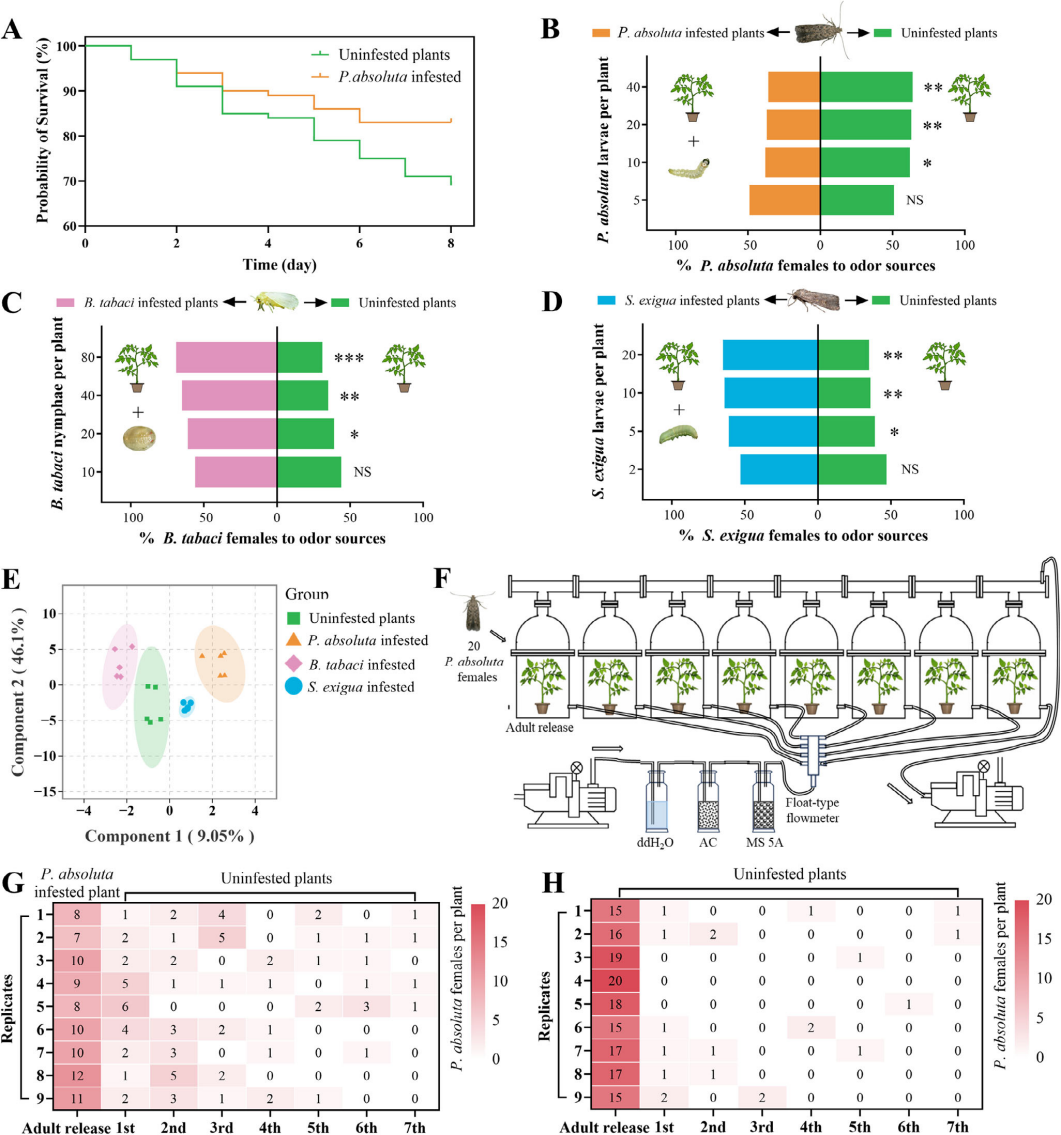
Effect of larval infestation on performance and the dispersal ability of *Phthorimaea absoluta*. (A) The Kaplan‐Meier survival curves of *P. absoluta* larvae fed on uninfested and *P. absoluta* larvae‐infested tomato plants during 8 days (*n* = 100 larvae). (B) Response of *P. absoluta* females to uninfested or larvae‐infested (5‐40/plant) tomato plants (*n* = 100 females) in Y‐tube olfactometers. Exact *p*‐values were as follows: 10 larvae (*χ* ^2^ = 5.76, *df* = 1, *p* = 0.016); 20 larvae (*χ* ^2^ = 6.76, *df* = 1, *p* = 0.009); 40 larvae (*χ* ^2^ = 7.84, *df* = 1, *p* = 0.005). (C) Response of *Bemisia tabaci* females to uninfested or nymph‐infested (10‐80/plant) tomato plants (*n* = 100 females) in Y‐tube olfactometers. Exact *p*‐values were as follows: 20 nymphs (*χ* ^2^ = 4.84, *df* = 1, *p* = 0.028); 40 nymphs (*χ* ^2^ = 9.00, *df* = 1, *p* = 0.003); 80 nymphs (*χ* ^2^ = 14.44, *df* = 1, *p* < 0.001). (D) Response of *Spodoptera exigua* females to uninfested or larval‐infested (2‐20/plant) tomato plants (*n* = 100 females) in Y‐tube olfactometers. Exact *p*‐values were as follows: 5 larvae (*χ* ^2^ = 4.84, *df* = 1, *p* = 0.028); 10 larvae (*χ* ^2^ = 7.84, *df* = 1, *p* = 0.005); 20 larvae (*χ* ^2^ = 9, *df* = 1, *p* = 0.003). (E) OPLS‐DA (Orthogonal Partial Least‐Squares Discriminant Analysis) principal component analysis of tomato plant HIPVs under attack by different herbivores (*n* = 5 plants). (F) Schematic of the *P. absoluta* female dispersal wind tunnel. (G) Dispersal of *P. absoluta* females on larvae‐infested tomato plants in the wind tunnel setup. (H) Dispersal of *P. absoluta* females on uninfested tomato plants in the wind tunnel setup. Survival curve comparison between treatments in (A) was analyzed using a Log‐Rank test. Y‐tube behavioral choice data in (B), (C), and (D) were analyzed using chi‐square tests. Asterisks indicate significant differences (^*^ *p* < 0.05, ^**^ *p* < 0.01, ^***^ *p* < 0.001). The count data following Poisson distributions in (G) and (H) were analyzed using General Linear Mixed Model (GLMM) analysis followed by LSD multiple range tests.

To identify the specific factors driving this shift in oviposition behavior, we hypothesized that odors released by feeding larvae or infested plants might repel ovipositing females. Therefore, we conducted Y‐tube olfactometer tests to examine the odor cues influencing adult female behavior. We found that odors from *P. absoluta* larvae (Chi‐square test: *χ* ^2^ = 0.036, *df* = 1, *p* = 0.549) and their frass (Chi‐square test: *χ* ^2^ = 0.400, *df* = 1, *p* = 0.841) did not affect the choice behavior of *P. absoluta* females (Figure S1C). Mechanical damage also did not affect female choice behavior (Chi‐square test: *χ* ^2^ = 0.321, *df* = 1, *p* = 0.571) (Figure S1C). However, plants infested with *P. absoluta* larvae for 24–60 h, but not before 24 h, were highly repellent to *P. absoluta* females (Figure S1C). This suggests that plant‐mediated signals deter females from laying eggs on infested plants, potentially motivating them to seek uninfested hosts.

To evaluate if the avoidance of herbivore damage is a general response of tomato pests, we conducted experiments comparing conspecific and heterospecific herbivore behavioral responses and plant volatile profiling changes under attack by three different herbivores: the chewing insects *P. absoluta* and *Spodoptera exigua*, and the piercing‐sucking insect, *Bemisia tabaci*. In the Y‐tube olfactometer and oviposition cage experiments, adults of the three herbivores showed different responses to tomato plants infested by their larvae/nymphs. Similar to the oviposition choice (Figure S1B), *P. absoluta* female adults were repelled by plants infested by 10, 20, and 40 *P. absoluta* larvae in the Y‐tube olfactometer (Figure 1B). In contrast, *B. tabaci* female adults were attracted to plants infested by 20, 40, and 80 *B. tabaci* nymphs in a Y‐tube olfactometer (Figure 1C), and laid more eggs on plants infested by *B. tabaci* nymphs in cage experiments (Figure S1G). Similarly, *S. exigua* female adults were also attracted to plants infested by 5, 10, and 20 *S. exigua* larvae in Y‐tube olfactometers (Figure 1D), and laid more eggs on plants infested by *S. exigua* larvae in cage experiments (Figure S1K). The oviposition choices of the three herbivores to heterospecific herbivore infestation were also investigated. In contrast to reduced oviposition of *P. absoluta* females by *P. absoluta* larvae infestations, *P. absoluta* larvae infestations did not affect the oviposition choices of *B. tabaci* and *S. exigua* females (Figure S1D,E). However, *B. tabaci* nymph and *S. exigua* larvae infestations increased the number of eggs laid by *P. absoluta* females (Figure S1F,I), as well as by *S. exigua* and *B. tabaci* adults (Figure S1H,J). These results imply that the repellency to *P. absoluta* female adults was specific for *P. absoluta* larvae infestation.

Volatiles of uninfested tomato plants and plants infested for 48 h by *P. absoluta* (10 second instar larvae/plant), *S. exigua* (5 second instar larvae/plant), or *B. tabaci* (20 nymphs/plant) were collected by a dynamic headspace collection system and analyzed by gas chromatography‐mass spectrometry (GC‐MS). Using Orthogonal Partial Least‐Squares Discriminant Analysis (OPLS‐DA), we observed a clear separation among the volatile profiles of uninfested, *P. absoluta*‐infested, *B. tabaci*‐infested, and *S. exigua*‐infested tomato plants (Figure 1E). A total of 33 compounds were detected in the headspace of uninfested tomato plants, whereas 35, 34, and 34 compounds were detected for *P. absoluta*‐, *B. tabaci*‐, and *S. exigua‐*infested tomato plants, respectively (Table S1). Furthermore, analysis based on OPLS‐VIP (Variable Importance in Projection) values revealed that benzaldehyde (2.528), β‐pinene (2.221), α‐phellandrene (1.811), 2‐carene (1.582), (Z)‐β‐ocimene (1.529), α‐pinene (1.523), methyl salicylate (1.230), and (Z)‐3‐hexenol (1.220) made significant contributions to the separations among the 4 treatments (a higher VIP value indicates that the corresponding compound plays a more important role in separation among treatments). These results imply that the different conspecific and heterospecific herbivore behavioral responses to tomato plants under attack by different herbivores were likely related to differences in HIPV emissions.

To investigate the effect of larval infestation on the adult dispersal ability of *P. absoluta*, a wind tunnel setup was designed (Figure 1F). When 20 *P. absoluta* females were released into the adult release jar (Figure 1F: jar 1) containing an uninfested tomato plant, an average of 2.33 females dispersed out of this jar into the adjacent 7 jars, which also contained uninfested plants (Figure 1H). In contrast, when the adult release jar contained a larvae‐infested plant, significantly more females (an average of 9.44) dispersed out of the adult release jar into the adjacent 7 jars containing uninfested plants (GLMM test: *χ* ^2^ = 113.080, *df* = 1, *p* < 0.001) (Figure 1G). These results imply that *P. absoluta* larval infestation elicited the release of HIPVs that repelled *P. absoluta* females and motivated their dispersal.

### 2.2 Identification of HIPVs That Trigger Dispersal of *P. absoluta* Females

Volatiles were collected from uninfested tomato plants and plants infested by *P. absoluta* larvae (10 second‐instar larvae for 48 h). GC‐MS analysis identified 35 compounds across both treatments (Table S2). Of these 35, 15 showed differential accumulations, with 11 significantly elevated and 4 significantly reduced in *P. absoluta*‐infested compared to uninfested plants (Figure 2A). Notably, (Z)‐3‐hexenol and benzaldehyde were specifically released from *P. absoluta*‐infested plants and were not detected in the headspace of uninfested plants (Figure 2A).

**FIGURE 2:**
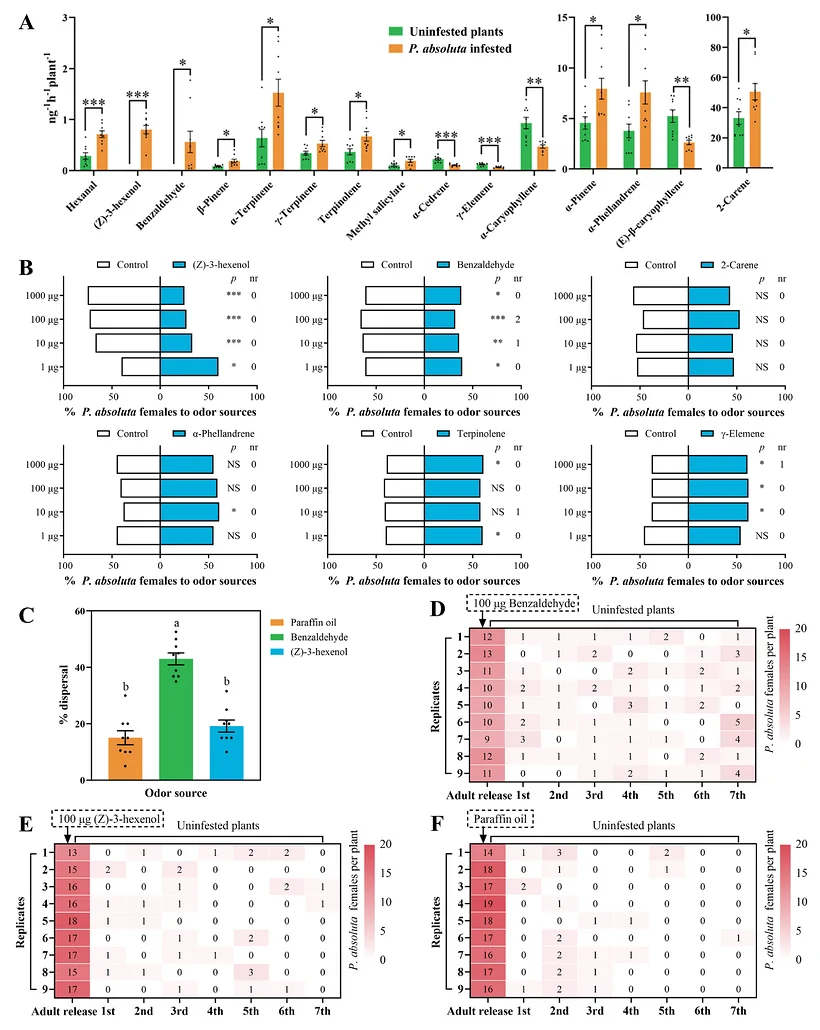
Identification of HIPVs that trigger *P. absoluta* female adult dispersal. (A) Comparative analysis of differentially accumulated volatile compounds in the headspace of uninfested and *P. absoluta* larvae‐infested tomato plants (*n* = 9 plants). (B) Responses of *P. absoluta* females in Y‐tube olfactometers to HIPVs at four doses (*n* = 100 adults). (C) The percentages of *P. absoluta* females dispersing from the adult release jar spiked with different odor sources (*n* = 9). Exact *p*‐values were <0.001 (paraffin oil vs benzaldehyde), <0.001 (benzaldehyde vs (Z)‐3‐hexenol), and 0.170 (paraffin oil vs (Z)‐3‐hexenol). The dispersal of *P. absoluta* females on uninfested tomato plants in Jar 1 spiked with: (D) 100 µg benzaldehyde (*n* = 9 replicates); (E) 100 µg (Z)‐3‐hexenol (*n* = 9 replicates); and (F) 10 µL paraffin oil (*n* = 9 replicates). Differences between means (±SE) in A) were analyzed using a two‐tailed unpaired *t*‐test. Y‐tube behavioral choice data in (B) were analyzed using chi‐square tests. The count data following Poisson distributions in (C–F) were analyzed using General Linear Mixed Model (GLMM) analysis followed by LSD multiple range tests. Asterisks indicate significant differences (^*^ *p* < 0.05, ^**^ *p* < 0.01, ^***^ *p* < 0.001), and different letters above bars indicate significant differences among treatments.

To test whether these specifically released HIPVs contribute to the repellency of *P. absoluta*, electroantennographic (EAG) responses of *P. absoluta* females to 15 HIPVs were determined using an EAG detector. The results showed that all 15 elicited EAG responses in *P. absoluta* females, with 6 HIPVs (Z)‐3‐hexenol, benzaldehyde, 2‐carene, α‐phellandrene, terpinolene, and γ‐elemene causing significantly elevated EAG responses (Figure S2). Behavioral responses of *P. absoluta* females in a Y‐tube olfactometer to these 6 were conducted at four doses. Benzaldehyde had a significant repellent effect compared to the control (hexane) at all four doses (1000 µg: *p =* 0.021; 100 µg: *p* < 0.001; 10 µg: *p =* 0.005; 1 µg: *p =* 0.028) (Figure 2B). (Z)‐3‐hexenol also showed a significant repellent effect, except at the lowest dose (1 µg), which significantly attracted *P. absoluta* females (1000 µg: *p* < 0.001; 100 µg: *p* < 0.001; 10 µg: *p* < 0.001; 1 µg: *p =* 0.046) (Figure 2B). Three compounds, α‐phellandrene (10 µg: *p =* 0.021), terpinolene (1000 µg: *p =* 0.028; 1 µg: *p =* 0.046), and γ‐elemene (1000 µg: *p =* 0.021; 100 µg: *p =* 0.016; 10 µg: *p =* 0.016), were significantly attractive to *P. absoluta* females at certain doses (Figure 2B). 2‐carene had no effect on the behavior of *P. absoluta* females (Figure 2B).

We next evaluated the effect of the two repellent compounds, (Z)‐3‐hexenol and benzaldehyde, on the dispersal of *P. absoluta* females in the wind tunnel. The percentage of *P. absoluta* females that dispersed from the adult release jar with different odor sources showed significant differences (Figure 2C, GLMM test: *χ^2^* = 100.733, *df* = 2, *p* < 0.001). When 20 *P. absoluta* females were released into the adult release jar (Figure 1F) with an uninfested tomato plant spiked with 100 µg benzaldehyde, 43.86% female adults (an average of 8.33 females) dispersed into the adjacent 7 jars with uninfested plants (Figure 2D), which was significantly higher than for the control (adult release jar with uninfested tomato plant and paraffin oil) (Figure 2F). In contrast, when 20 *P. absoluta* females were released into the adult release jar with an uninfested tomato plant spiked with 100 µg (Z)‐3‐hexenol, only 16.67% female adults (an average of 3.33 females) dispersed into the adjacent jars (Figure 2E), which was not significantly different from the control (Figure 2E). These results imply that benzaldehyde is the key larvae‐feeding‐induced volatile that triggers *P. absoluta* dispersal.

### 2.3 Identification of Infestation‐Induced Benzaldehyde‐Release‐Related Genes and Stomatal Measurements in Tomato

The release of plant volatiles, including HIPVs, is generally thought to be controlled by their biosynthesis rate, as well as stomatal conductance [17, 47]. To identify genes related to benzaldehyde release in response to *P. absoluta* larvae infestation, gene expression analysis of benzaldehyde biosynthesis‐related genes was carried out using previously published RNA‐seq data [48].

Surprisingly, the transcript levels of all 16 identified benzaldehyde biosynthesis related genes, including PAL2 (phenylalanine ammonia lyase 2), PAL3 (phenylalanine ammonia lyase 3), PAL5 (phenylalanine ammonia lyase 5), LOC101243656, LOC101261892, LOC112941051, LOC112941051, LOC101263745, LOC101265549, LOC101246396, alpha‐DOX2 (alpha‐dioxygenases 2), CCR1 (cinnamoyl‐CoA reductase 1), CCR2 (cinnamoyl‐CoA reductase 2), LOC101250958, LOC109120859, LOC101268681, and LOC101250164 gene, were not significantly different between uninfested and P. absoluta‐infested tomato plants (|log_2_(fold change) | ≥ 1 and *p* < 0.05) (Figure S3A).

Since benzaldehyde is reported to be toxic to plant cells and can be readily reduced to benzyl alcohol [49], we hypothesized that the increased release of benzaldehyde following *P. absoluta* infestation might be attributed to changes in its release pathway rather than its biosynthesis pathway. To test this inference, we investigated the total volatile release, the stomatal aperture of tomato leaves, and the expression of stomata‐related genes. The total volatiles released from plants (*t‐*test: *t* = 2.905, *df* = 16, *p* = 0.010) and the stomatal apertures of tomato leaves (*t‐*test: *t* = 16.336, *df* = 98, *p* < 0.001) were significantly increased after *P. absoluta* larvae infestation (Figure 3A–C). Moreover, in 140 identified stomata related genes (Figure S3B–E), the transcript levels of 7 genes (up: *LOC101245524*, *LOC101265748*, *LOC101262338*, *LOC101256903*; down: *LOC101255217*, *LOC101250825*, *LOC101256653*) were significantly altered in *P. absoluta*‐infested tomato leaves (Figure 3D). Among these, *LOC101245524* and *LOC101265748*, encoding IQ‐Motif Containing Proteins *IQM1* and *IQM4*, respectively, were the most significantly differentially expressed between uninfested and *P. absoluta‐*infested tomato plants.

**FIGURE 3:**
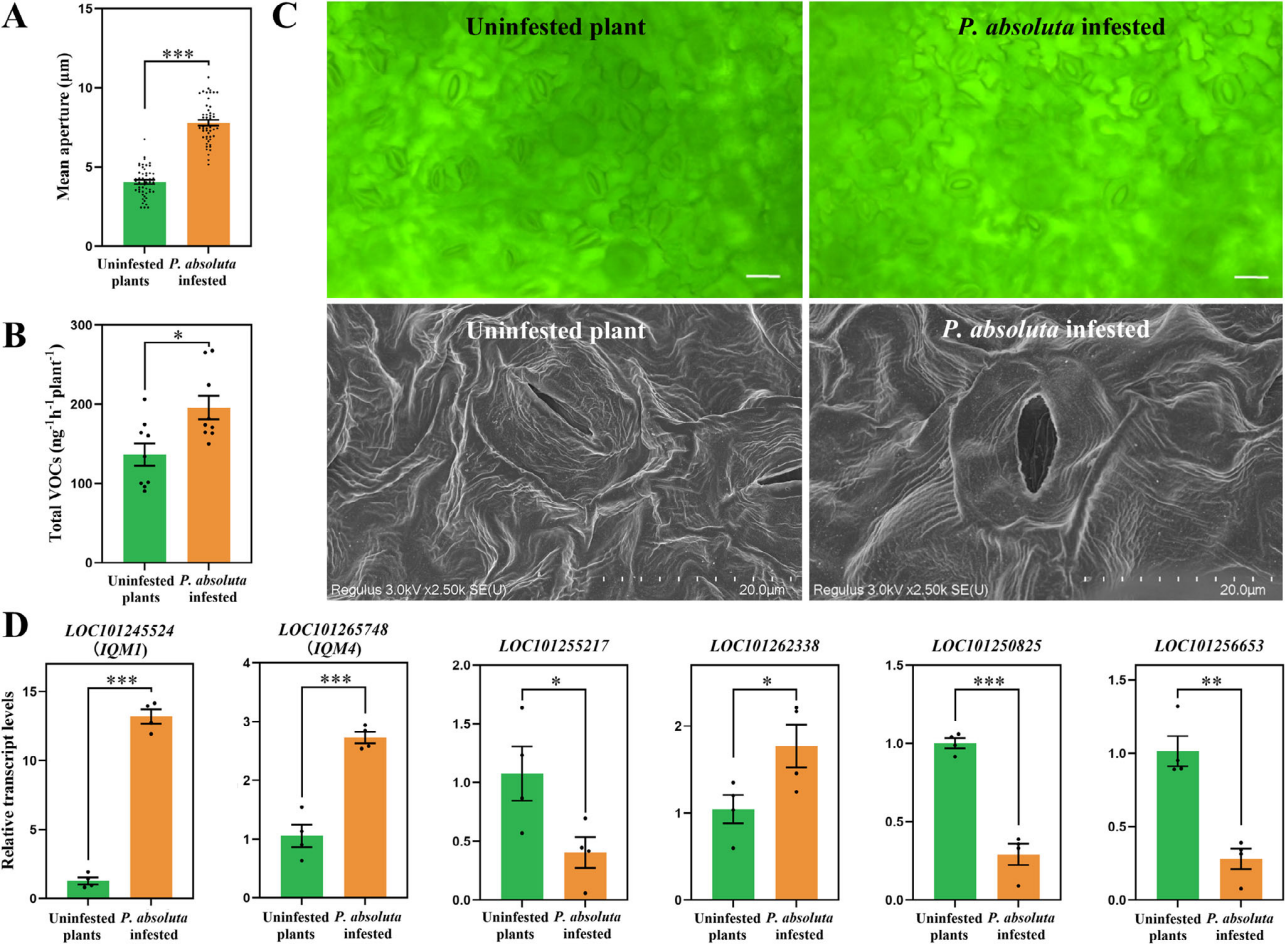
The effect of *P. absoluta* larvae infestation on total volatile release, stomatal apertures of tomato leaves, and transcript levels of stomatal‐related genes. (A) Total VOCs released by uninfested and *P. absoluta*‐infested tomato plants (*n* = 9 plants). (B) The stomatal aperture of tomato leaves from uninfested and *P. absoluta*‐infested tomato plants (*n* = 50 stomata). (C) Representative images of leaf stomata of uninfested and *P. absoluta*‐infested tomato plants from light (upper, bars 20 µm) and electron microscopy. (D) Transcript levels of stomatal‐related genes in uninfested and *P. absoluta*‐infested tomato plants (*n* = 4 biological replicates). For transcript levels, exact *p*‐values were as follows: *LOC101245524* (*t* = 20.400, *df* = 6, *p* < 0.0001), *LOC101265748* (*t* = 7.726, *df* = 6, *p* = 0.0002), *LOC101255217* (*t* = 2.534, *df* = 6, *p* = 0.044), *LOC101262338* (*t* = 2.455, *df* = 6, *p* = 0.049), *LOC101250825* (*t* = 9.434, *df* = 6, *p* < 0.0001), *LOC101256653* (*t* = 5.913, *df* = 6, *p* = 0.001), *LOC101256903* (*t* = 3.323, *df* = 6, *p* = 0.039). Differences between means (±SE) in A), B), and D) were analyzed using a two‐tailed unpaired *t*‐test. Asterisks indicate significant differences (^*^ *p* < 0.05, ^**^ *p* < 0.01, ^***^ *p* < 0.001).

To assess whether *IQM1*‐mediated stomatal opening is specifically triggered by *P. absoluta* infestation, we examined the effects of two other tomato pests, the chewing insect *S. exigua* and the phloem‐feeding *B. tabaci*, on stomatal aperture and *IQM1* expression in wild‐type tomato plants (Figure S4A–E). In contrast to the stomatal opening and upregulated *IQM1* expression observed in response to *P. absoluta* (Figure 3B–D), infestation by *B. tabaci* affected neither stomatal aperture (Figure S4A,B) nor *IQM1* expression (Figure S4D). Meanwhile, *S. exigua* infestation induced stomatal closure (Figure S4A,C) without altering *IQM1* transcript levels (Figure S4E). These findings suggest that stomatal responses are herbivore‐specific and that both *IQM1* upregulation and stomatal opening are specifically associated with *P. absoluta* attack.

### 2.4 Functional Analysis of *IQM1* Using VIGS

To examine the roles of *IQM1* in stomata opening, VOCs release, and *P. absoluta* performance, the function of *IQM1* was tested using a virus‐induced gene silencing (VIGS) technique. When the VIGS tomato plants reached the 5‐fully‐expanded‐leaf stage (Figure S5), the transcript levels of *IQM1*, stomatal apertures, and total VOCs release were assessed in VGFP and VIQM1 plants. Relative to VGFP plants, *IQM1* transcript levels, stomatal apertures, and total VOCs released were significantly reduced in VIQM1 plants (Figure 4C–F). After *P. absoluta* infestation, *IQM1* transcript levels, stomatal apertures, and total VOCs released were significantly increased in VGFP plants (Figure 4C–F). In contrast, in VIQM1 plants, the induced increase in *IQM1* transcripts by *P. absoluta* infestation was suppressed (Figure 4C). Moreover, silencing of the *IQM1* gene by VIGS suppressed the infestation‐induced increase in stomatal apertures (Figure 4D,F) and the total VOC release (Figure 4E).

**FIGURE 4:**
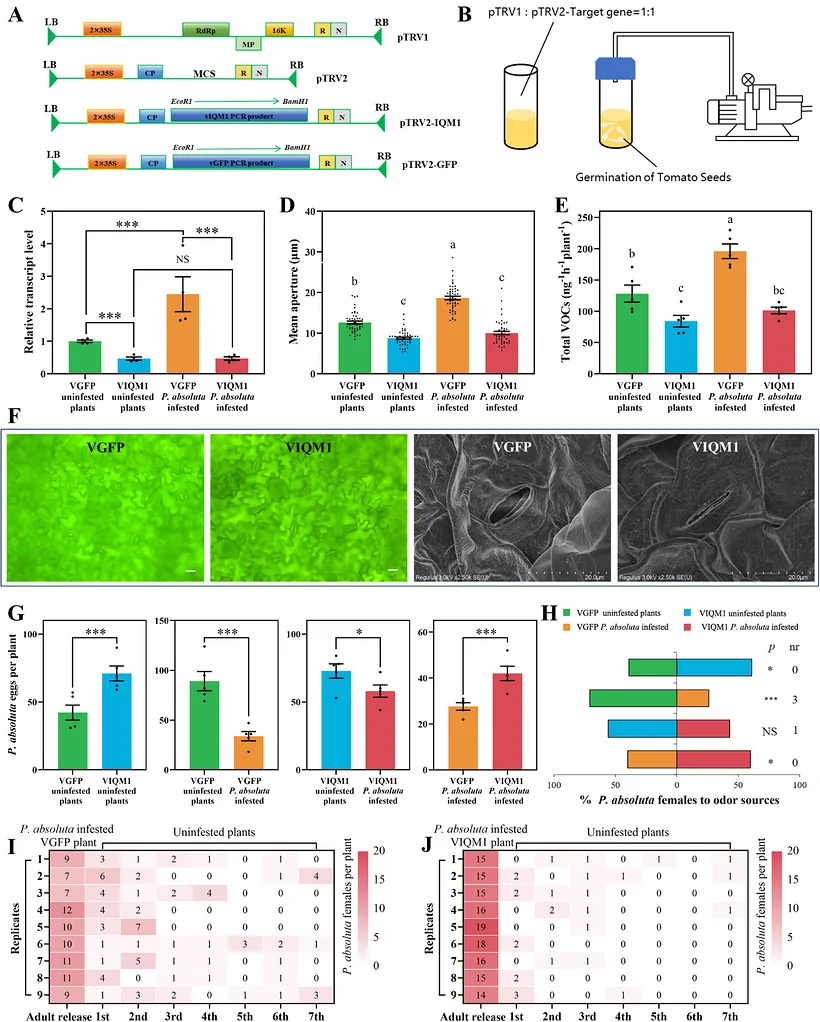
Effect of *IQM1* silencing on infested tomato transcripts, stomatal apertures and VOCs release, and *Phthorimaea absoluta* oviposition, preference and dispersal. (A) Plasmid structure of TRV‐based virus‐induced gene silencing (VIGS) vectors; (B) VIGS seed infection system; (C) The transcript levels of the *IQM1* gene 25 days after VIGS treatment. Asterisks indicate significant differences among the 4 treatments (*n* = 4 plants/treatment). For *IQM1* gene expression, exact *P*‐values of VGFP uninfested plants vs VIQM1 uninfested plants (*t* = 8.869, *df* = 6, *p* < 0.001), VGFP uninfested plants vs VGFP *P. absoluta* infested plants (*t* = ‐2.686, *df* = 6, *p* = 0.036), VIQM1 uninfested plants vs VIQM1 *P. absoluta* infested plants (*t* = ‐0.041, *df* = 6, *p* = 0.968), VGFP *P. absoluta* infested vs VIQM1 *P. absoluta* infested plants (*t* = 3.661, *df* = 6, *p* = 0.011). (D) The effect of *IQM1* gene silencing on the stomatal diameter of tomato leaves (*n* = 50 stomata). Exact *p*‐values: one‐way ANOVA, Tukey's HSD test, *F*_3,196_ = 135.991, *p* < 0.0001. (E) The effect of *IQM1* gene silencing on the release of total plant volatiles in tomato plants (*n* = 5 plants/treatment). Exact *P*‐values: one‐way ANOVA, Tukey's HSD test, *F*_3,16_ = 22.210, *p* < 0.0001. (F) Representative images of leaf stomata of VGFP and VIQM1 plants from light (left, bars 20 µm) and electron microscopy (right). (G) The effect of *IQM1* gene silencing on the oviposition preference of *P. absoluta* females (*n* = 5 plants/treatment). exact *p*‐values of VGFP uninfested plants vs VIQM1 uninfested plants (*χ* ^2^ = 16.986, *df* = 1, *p* < 0.001), VGFP uninfested plants vs VGFP *P. absoluta* infested plants (*χ^2^* = 33.136, *df* = 1, *p* < 0.0001), VIQM1 uninfested plants vs VIQM1 *P. absoluta* infested plants (*χ^2^* = 5.688, *df* = 1, *p* = 0.017), VGFP *P. absoluta* infested vs VIQM1 *P. absoluta* infested plants (*χ^2^* = 20.474, *df* = 1, *p* < 0.0001). H) The effect of *IQM1* gene silencing on behavioral response of *P. absoluta* female adults in Y‐tube assays (*n* = 100 adults in total). exact *p*‐values of VGFP uninfested plants vs VIQM1 uninfested plants (*χ^2^* = 4.840, *df* = 1, *p* = 0.028), VGFP uninfested plants vs VGFP *P. absoluta* infested plants (*χ^2^* = 20.455*, df* = 1, *p* < 0.0001), VIQM1 uninfested plants vs VIQM1 *P. absoluta* infested plants (*χ^2^ =* 1.707, *df =* 1, *p =* 0.191), VGFP *P. absoluta* infested vs VIQM1 *P. absoluta* infested plants (*χ^2^* = 4.000, *df* = 1, *p* = 0.046). (I) Wind tunnel dispersal of *P. absoluta* females on *P. absoluta* larvae infested VGFP plants (*n* = 9 experimental replicates). (J) Wind tunnel dispersal of *P. absoluta* females on *P. absoluta* larvae infested VIQM1 plants (*n* = 9 experimental replicates). Differences among means (±SE) in C) were analyzed using a two‐tailed unpaired *t*‐test. Differences among means (±SE) in (D and E) were analyzed using one‐way ANOVA followed by Tukey's HSD test. Significance between treatments in (G) was determined by a two‐sided likelihood ratio test with generalized linear Mixed models (binomial distribution error); Y‐tube behavioral choice data in (H) were analyzed using chi‐square tests. Asterisks indicate significant differences (^*^ *p* < 0.05, ^**^ *p* < 0.01, ^***^ *p* < 0.001). Different letters indicate significant differences among treatments.

We next evaluated the effect of silencing *IQM1* on *P. absoluta* performance. Relative to VGFP uninfested plants, VIQM1 uninfested plants were more attractive to *P. absoluta* adults in Y‐tube assays and received more frequent oviposition (Figure 4G,H), this baseline difference may reflect a subtle change in constitutive volatile emission or leaf surface properties of VIQM1 plants. Infested VGFP plants repelled *P. absoluta* adults and decreased ovipositions (Figure 4G,H). In contrast, with VIQM1 plants, these induced repellent effects disappeared (Figure 4H), or were attenuated (Figure 4G). Relative to infested VGFP plants, infested VIQM1 plants were more attractive to *P. absoluta* adults in Y‐tube assays and received a greater number of ovipositions (Figure 4G and H). In addition, the dispersal of *P. absoluta* adults from infested VGFP and VIQM1 plants in the wind tunnel revealed fewer adults dispersing from VIQM1 plants (2.778 adults) than from VGFP plants (8.556 adults) (likelihood ratio (LR) test with GLMM, *χ^2^* = 97.344, *df* = 1, *p* < 0.0001) (Figure 4I,J).

### 2.5 Knocking Out *IQM1* Attenuates HIPV Release, *P. absoluta* Dispersal and Population Growth

The function of *IQM1* was further examined by generating loss‐of‐function mutants in cultivated tomato (cv. ZF202) using CRISPR‐Cas9 genome editing (Figure 5A). Homozygous IQM1 mutant (MT2‐1) and wild‐type (cv. ZF202) tomato plants were used to analyze VOCs release, insect performance (via life‐table analyses), adult dispersal ability, and population growth of *P. absoluta* in large‐scale glasshouse experiments. Comparison of plant height, stem diameter, and chlorophyll content between wild‐type and *IQM1* mutant tomato plants at the age of 25, 35, and 45 days old showed that knocking out *IQM1* did not affect plant growth (Figure S6). Knocking out *IQM1* attenuated *P. absoluta*‐induced stomatal opening, without affecting *B. tabaci‐* and *S. Exigua‐*induced stomatal responses (Figures S4 and S7). Knocking out *IQM1* significantly attenuated *P. absoluta*‐induced HIPV release (Figure 5B,C; Figure S8). OPLS‐DA of detected volatiles showed a clear separation along axis I between infested and control WT tomato plants (Figure 5B) (the OPLS‐VIP value of Z‐3‐hexenol, benzaldehyde, hexanal, α‐cedrene, and α‐caryophyllene was 2.060, 1.753, 1.583, 1.549, and 1.393, respectively), while the distinction between infested and control plants of the mutant line MT2‐1 was highly attenuated (Figure 5B). Total volatiles released increased dramatically after infestation in wild‐type plants (Figure 3B). but did not change significantly when plants of the *IQM1* mutant line (MT2‐1) were infested (Figure 5C, *t* = ‐1.273, *df* = 16, *p* = 0.221). Moreover, the majority of the differentially accumulating volatiles between control and infested wild‐type plants (Table S2), most notably benzaldehyde, no longer differed in infestations of the *IQM1* mutant line, except for hexanal and (Z)‐3‐hexenol (Figure 5C, Tables S2 and S3). To compare *P. absoluta* performance on WT and MT2‐2 plants, a complete life table analysis was performed (Table S7). No statistical differences were found in egg duration and survival, larval duration, male and female pupal duration, adult survival, longevity, total fecundity, and sex ratios. Only a marginally higher (p = 0.046) percentage larval survival on MT2‐2 plants was found (Table S7).

**FIGURE 5:**
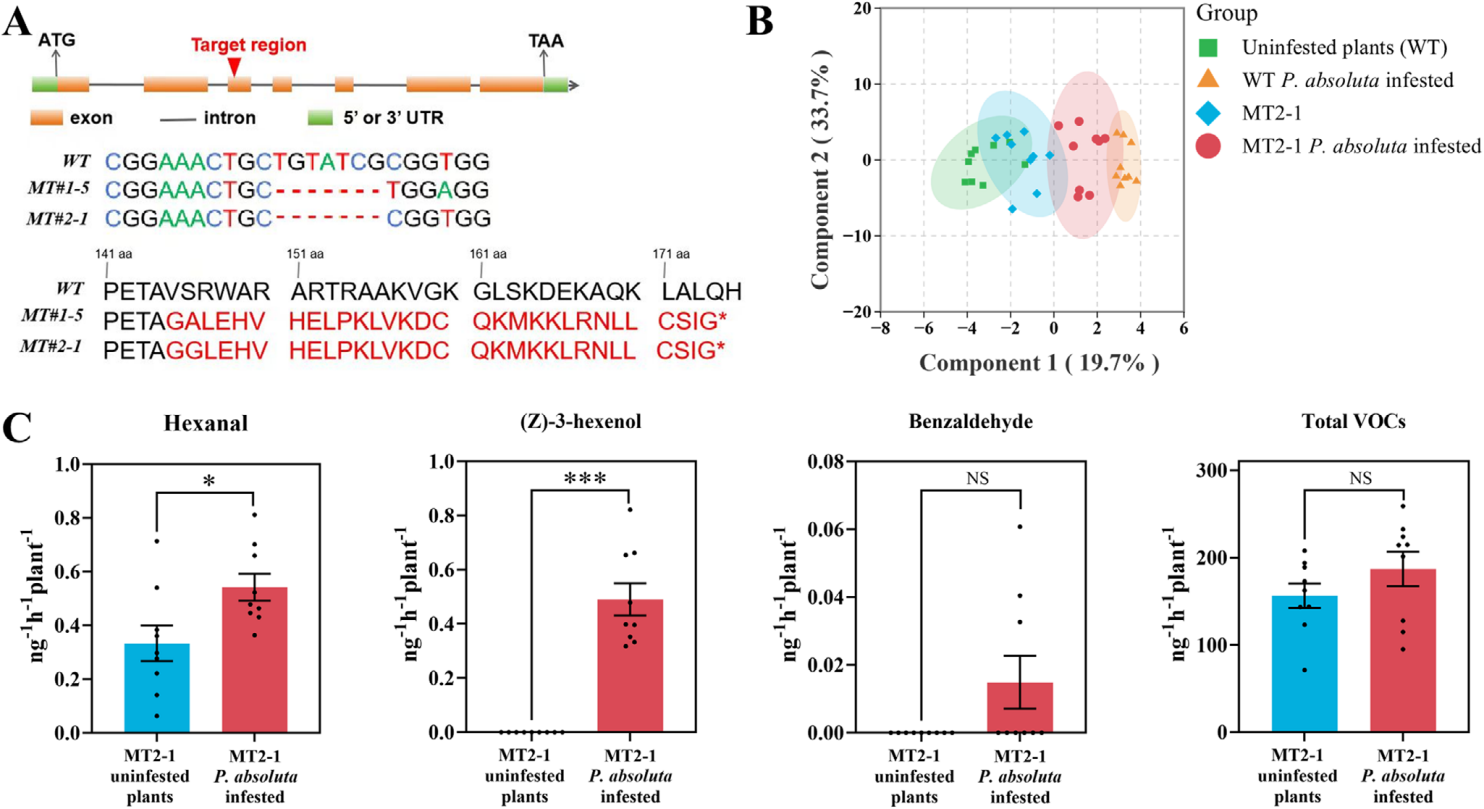
CRISPR‐Cas9‐mediated knockout of *IQM1* results in changes in larvae feeding‐induced volatiles release of tomato. (A) Schematic representation of *IQM1* with location of guide RNA sequences, CRISPR‐Cas9 cleavage sites, and *IQM1* mutant sequences. Two independent, homozygous *IQM1* mutant lines (#1 and #5) were generated in a tomato (cv. ZF202) background. (B) OPLS‐DA (Orthogonal Partial Least‐Squares Discriminant Analysis) principal component analysis of HIPVs of wild‐type (WT) and MT2‐1 tomato plants infested by *P. absoluta*. (C) Comparative analysis of differentially accumulated volatile compounds (Hexanal, (Z)‐3‐hexenol, and benzaldehyde) and total VOCs between uninfested and larvae‐infested IQM1 mutant line (MT2‐1) (*n* = 7–9 plants). Differences between means (±SE) in C) were analyzed using a two‐tailed unpaired *t*‐test. Asterisks indicate significant differences (^*^ *p* < 0.05, ^**^ *p* < 0.01, ^***^ *p* < 0.001).

Simulated field assays were conducted to evaluate population dynamics and dispersal patterns of *P. absoluta* on wild‐type and *IQM1* mutant plants for two generations in glasshouse experiments (Figure 6A; Figure S9). In the F_1_ generation, the population trend index (*I*) decreased in populations of *IQM1* mutant plants compared to that of wild‐type populations, although the difference was not statistically significant (*t* = 1.372, *df* = 4, *p =* 0.241) (Figure 6B). While in the F_2_ generation, this decrease was significantly lower (*t* = 3.332, *df* = 4, *p* = 0.029; Figure 6C). Similar trends were seen in the indices of patchiness (Kuno's *C*_A_), which tended to increase in the F_1_ generation in populations of the mutant plants (*t* = 1.908, *df* = 4, *p* = 0.129; Kuno's *C*_A‐wt_ = 0.69 ± 0.347; Kuno's *C*_A‐mutant_ = 1.67 ± 0.377; Figure 6D) and became significantly higher in the F_2_ generation (*t* = 4.77, *df* = 4, *p* = 0.009; Kuno's *C*_A‐wt_ = 0.24 ± 0.062; Kuno's *C*_A‐mutant_ = 1.62 ± 0.281; Figure 6E). Clearly visible patterns of *P. absoluta* aggregation were observed in populations of the *IQM1* mutant line compared to wild‐type tomato plants (Figure 6F,G; Figure S10). By the end of the F_2_ generation in experimental populations of *IQM1* mutant plants, *P. absoluta* populations on some tomato plants had exhausted their food supply, resulting in large numbers of larval deaths (Figure S11). Thus, aggregation (high Kuno's *C*_A_) on mutant plants directly reflects the failure of benzaldehyde‐mediated dispersal, while low aggregation on wild‐type plants indicates successful redistribution of females away from the initially infested plant. These results reveal that a knock‐out of *IQM1*, despite its beneficial effects on larval survival (Table S7), significantly decreased population growth and reduced dispersal behavior of *P. absoluta*, results which are consistent with the effects of *IQM1* on HIPV release.

**FIGURE 6:**
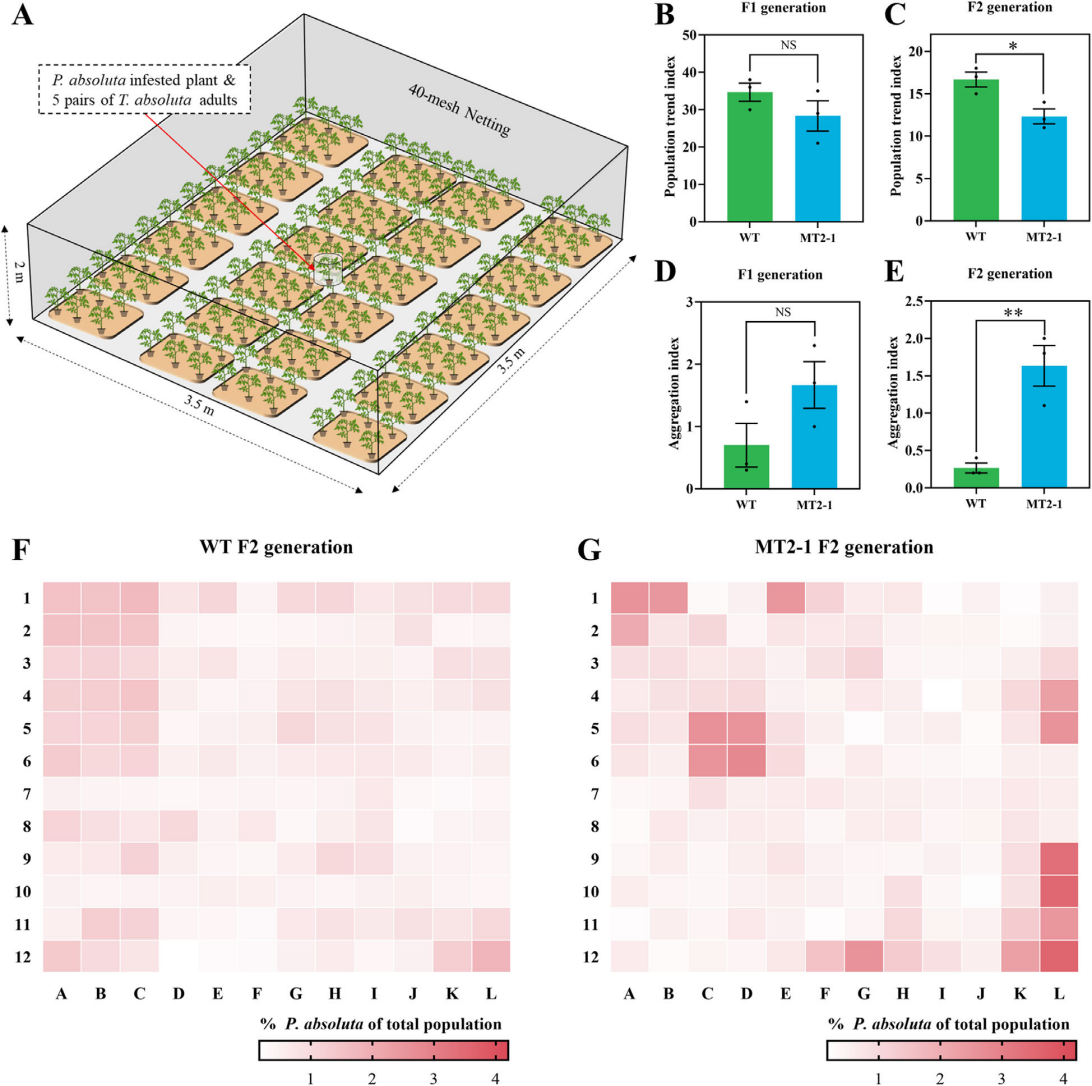
Population dynamics and dispersal patterns of *P. absoluta* on wild‐type and IQM1 plants. (A) Schematic of dispersal and population growth experiments conducted in large cages in a glasshouse. (B) and (C) Population trend indices (*I*) of *P. absoluta* in F_1_ and F_2_ generations (*n* = 3). (D) and (E) Indices of patchiness (Kuno's *C*_A_) of *P. absoluta* in F_1_ and F_2_ generations (*n* = 3). (F) and (G) Population aggregation patterns of *P. absoluta* in the F_2_ generation on wild‐type (F) and IQM1 mutant plants (G) in one of the three experimental replicates (see Figure S10 for the other replicates). Differences between means (±SE) in (B–E) were analyzed using two‐tailed unpaired *t*‐tests. Asterisks indicate significant differences (^*^ *p* < 0.05, ^**^ *p* < 0.01, ^***^ *p* < 0.001).

## 3 Discussion

As one of the most important structures in plants, stomata play an essential role in plant responses to biotic stresses, including herbivores and phytopathogens [18]. While existing knowledge on the roles of stomata is centered on plant‐pathogen interactions, the roles of stomata in plant‐herbivore interactions remain largely unknown. A few studies have shown that herbivores could manipulate stomatal apertures, with stomatal closure being a typical response, affecting HIPV emissions [19, 20, 21, 22, 23]. However, no studies have reported herbivory‐induced stomatal opening and HIPV release. In this study, we report that in tomato plants attacked by the invasive and outbreaking leafminer, *P. absoluta*, larval infestation induced the release of repellent HIPV, benzaldehyde, which was triggered by stomatal opening, rather than HIPV biosynthesis. These do not occur when tomato plants are attacked by two other tomato pests, *B. tabaci* and *S. exigua*. The ability of this herbivore to elicit stomatal opening, much as some pathogens do, illustrated a new pattern in stomata‐herbivore interactions, adding another layer of complexity to stomata‐mediated plant‐herbivore interactions. Further investigations on induced stomatal dynamics by various herbivore species are needed to gain further insight into stomata‐mediated plant‐herbivore interactions.

When further exploring the molecular mechanisms, we found that the *P. absoluta* infestation‐induced benzaldehyde release occurred through *IQ‐Motif Containing Protein 1* (*IQM1*) regulated stomatal openings, rather than the benzaldehyde biosynthesis pathway. The *IQM* is a novel IQ‐motif‐containing Calmodulin‐binding protein (CaMBP) family, with each *IQM* gene potentially playing a unique role in plant development and its response to environmental cues [50]. *IQM1* was found to be a Ca^2+^‐independent CaMBP that modulates stomata movement by affecting reactive oxygen species (ROS) content in *Arabidopsis* [51], which is consistent with *IQM1* regulating stomatal movement in tomato plants in this study. The benzaldehyde levels in plants were regulated through various pathways, including biosynthesis from trans‐cinnamic acid, release from hydrolysis of glycoside precursors, and conversion into downstream chemicals [52]. Benzaldehyde can be stored in glycosylated form (e.g., as benzyl alcohol glycosides) in plant cells and released upon hydrolysis by β‐glucosidases [49]. It is possible that the induced benzaldehyde in this study is from glycoside precursors rather than de novo synthesis. Future work is needed to investigate whether herbivory affects hydrolysis of glycoside precursors to regulate the release of benzaldehyde.

Stomata responses to biotic stresses are sophisticated and dynamic processes regulated by signaling networks. In contrast to the literature on the detailed molecular basis of stomata‐pathogen interactions [32, 33, 35], there are only a few reports on the molecular mechanism of stomata‐herbivore interactions [18, 27]. Plant‐herbivore interactions start with molecular recognition responses, in which herbivore‐associated molecular patterns (HAMPs) play important roles [17]. Many compounds in insect oral secretions are potential modulators of plant stomatal dynamics. For instance, salivary glucose oxidase (GOX) of *Helicoverpa zea* triggers stomatal closure for at least 48 h and inhibits the emissions of HIPVs in 24 h [27]. An effector, REPAT38‐like, from the oral secretions of *P. absoluta* larvae was involved in regulating stomatal conductance in tomato leaves [53]. In this study, compared with control third‐instar larvae, feeding by REPAT38 silenced third‐instar larvae significantly increased the stomatal opening ratio at 0.5 h, but decreased the stomatal opening ratio at 1 h [53], which stands in contrast with our results. This suggests that the regulation of stomatal conductance by HAMPs is as complicated and dynamic as that in stomata‐pathogen interactions. Previous work has shown that the influence of HAMPs (e.g., GOX) on stomata varied among plant species [27]. Other factors, such as larval stage and feeding time, might also contribute to the different results. Identification of the *P. absoluta* HAMPs that interact with stomatal‐control genes is an important goal for future research.

Changes in stomatal dynamics and HIPVs release can have ecological impacts on plants and herbivores. It has been hypothesized that herbivory‐induced stomatal closure may have benefits for herbivores, including maintaining plant water contents and increasing its nutritional quality to herbivores, increasing leaf temperature to facilitate herbivore growth, reducing competition by preventing invasion of plant pathogens, and inhibiting emission of plant defense‐related VOCs to reduce negative inter/intra species interactions to herbivores [18]. Here, we provide evidence that herbivory‐induced stomatal opening is also beneficial to herbivores. We found that *P. absoluta* larvae perform better on larvae‐infested tomato plants, while female moths are repelled from the larvae‐infested plants by induced releases of benzaldehyde, one of the most widely distributed volatiles produced by plants and arthropods that also plays important roles in insect chemical communication [54, 55]. This result is inconsistent with the “mother knows best” principle, which states that female insects will evolve to oviposit on hosts where their offspring fare best [56]. Why did *P. absoluta* females prefer to lay eggs on undamaged plants rather than larvae‐infested plants on which their larvae perform better? The results from the female dispersal experiment and large‐scale glasshouse trials provided a plausible explanation, showing that larvae‐infested plants motivate female adults to disperse and colonize uninfested plants, triggering the dispersal and population expansion of this invasive pest. Due to induced dispersal ability, *P. absoluta* population growth and outbreaks were amplified in glasshouse populations of wild‐type plants compared to populations of *IQM1*‐mutated plants, despite the greater survivorship of larvae on *IQM1*‐mutated plants. The contradiction between survivorship of larvae in life table analysis and population index (*I*) might be due to the reduced dispersal behavior of adult females on *IQM1*‐mutated plants. On *IQM1*‐mutated plants, the lack of induced benzaldehyde release reduced adult dispersal, which resulted in overcrowded damage of larvae on the same plants, increased larval mortality (Figure S11), and ultimately lower population growth. Without benzaldehyde‐mediated redistribution, females oviposit on or near the already infested plant, generating density‐dependent larval mortality that outweighs individual‐level benefits. Kuno's *C*_A_ index thus serves as an inverse proxy for dispersal. By comparing the conspecific and heterospecific herbivore behavioral responses of three different herbivores (*P. absoluta*, *S. exigua*, and *B. tabaci*) on tomato, we found that the manipulation of tomato plant defenses to avoid conspecific damage is specific for *P. absoluta*. The results represent another means by which insect invaders can leverage plant defense responses for their own benefits [57, 58, 59].

## 4 Experimental Section

### 4.1 Insects and Plants

The *P. absoluta* colony was collected from tomato in 2018 in Yili, Xinjiang, and was maintained on tomato plants (*Solanum lycopersicum cv*. ZF202) in cages in a climate chamber (25 ± 1°C, 60% ± 5% RH, 16 h: 8 h L: D photoperiod). The *Bemisia tabaci* MED colony was originally collected from tomato in 2022 in Hangzhou, Zhejiang, and was subsequently maintained on tomato plants (*S. lycopersicum cv*. ZF202) in cages in a climate chamber (25 ± 1°C, 60% ± 5% RH, 16 h: 8 h L: D photoperiod). The *Spodoptera exigua* colony was originally collected from pepper in 2023 in Hangzhou, Zhejiang, and was reared on an artificial diet in a climate chamber (25 ± 1°C, 60% ± 5% RH, 16 h: 8 h L: D photoperiod). Tomato (*S. lycopersicum cv*. ZF202) cultivation followed previously described procedures [48]. Plants with five fully expanded leaves were used for experiments, which occurred about 6 weeks after sowing.

### 4.2 *Phthorimaea absoluta* Larval Performance and Adult Dispersal Assays

Performance of *Phthorimaea absoluta* (Lepidoptera: Gelechiidae: Gelechiinae: Gnorimoschemini) larvae on uninfested and larvae‐infested tomato leaves was evaluated with excised leaf assays. Excised tomato leaves were infested with one second‐instar *P. absoluta* larva per leaf for 48 h, then the larvae were removed from the leaves. Uninfested tomato leaves were used as controls. A piece of an uninfested tomato leaf or a larvae‐infested tomato leaf was placed in Petri dishes (9.0 cm diam., 1.5 cm high) containing filter paper. The petioles were wrapped with absorbent cotton soaked in water. Five newly laid eggs were placed on each tomato leaf, with 20 replicates for each treatment. When the eggs hatched, the survivorship of larvae was observed every day, and the leaves in Petri dishes were changed daily with new uninfested or larvae‐infested tomato leaves. The pupation rate was also recorded for the two treatments.

Oviposition preferences of *P. absoluta* female adults on uninfested and larvae‐infested tomato plants were evaluated in cage experiments. Larvae‐infested tomato plants were obtained by infesting tomato plants with 10 second‐instar *P. absoluta* larvae per plant for 48 h, and then the larvae were removed from plants (the same for olfactory behavior and adult dispersal bioassays below). One uninfested tomato plant and one larvae‐infested tomato plant were placed in the two opposite corners of the insect rearing cage (50 × 50 × 50 cm, Nylon mesh with 300‐µm mesh, Ningbo Jiangnan Instrument Factory, Ningbo, Zhejiang). Twenty pairs of 2‐day‐old *P. absoluta* female and male adults were released in the middle of the cage. After 72 h, the number of *P. absoluta* eggs on all leaves of each tomato plant was counted. The experiment was repeated 11 times.

Behavioral responses of *P. absoluta* females to different odor sources were tested using Y‐tube olfactometers (6 cm stem; 6 cm arms at 90° angle; 1 cm internal diameter). Odor sources included clean air, volatiles from 10 second‐instar larvae dissected from tomato leaves, volatiles from 0.2 g fresh frass, volatiles from an uninfested tomato plant, and volatiles from tomato plants infested by 10 second‐instar *P. absoluta* larvae for 4 h, 8 h, 12 h, 18 h, 24 h, 36 h, 48 h, 60 h, 72 h, respectively, after which the larvae were removed from plants. One hundred *P. absoluta* females were tested for each pair of odor sources. The olfactory behavior assays were conducted using the method described by Chen et al. (2023) [60].

The conspecific herbivore behavioral responses under attack by three different herbivores, chewing insects *P. absoluta* and *Spodoptera exigua*, and piercing‐sucking insect *Bemisia tabaci*, were conducted using Y‐tube olfactometers. For *P. absoluta*, tomato plants infested by 5, 10, 20, and 40 second‐instar larvae for 48 h were used as odor sources. For *B. tabaci*, tomato plants infested by 10, 20, 40, and 80 nymphs for 48 h were used as odor sources. For *S. exigua*, tomato plants infested by 2, 5, 10, and 20 second‐instar larvae for 48 h were used as odor sources. In all three species, uninfested tomato plants were used as control odor sources, and the herbivores were removed prior to bioassays. Olfactometer bioassays were conducted as detailed above. The oviposition choices of three herbivores to conspecific and heterospecific herbivore infestation were also investigated in cage experiments as described above. Larvae/nymph‐infested tomato plants were obtained by infesting tomato plants with 10 second‐instar *P. absoluta* larvae, 20 *B. tabaci* nymphs, and 5 second‐instar *S. exigua* larvae per plant for 48 h, respectively. Uninfested tomato plants were used as controls. The number of eggs laid by *P. absoluta* (*n* = 20 adults/cage), *B. tabaci* (*n* = 40 adults/cage), and *S. exigua* adults (*n* = 10 adults/cage) was recorded for each combination after 72 h.

The effects of *P. absoluta* larvae infestation on the dispersal ability of female adults were tested in a wind tunnel setup (Figure 1E). The setup was composed of 8 connected polymethyl methacrylate jars. The components were connected with screws and sealed with parafilm film. The first jar was the adult release jar. Air was pulled into the setup and filtered sequentially through double‐distilled water, activated charcoal, and molecular sieves (5 A°, beads, 8–12 mesh, Sigma‐Aldrich) before entering the glass jars. The airflow was split into 8 jars (500 mL/min in each jar controlled by float‐type flowmeters) and pumped through the wind tunnel. The system was purged for 30 min with purified air before odor source plants were placed. An uninfested tomato plant or a tomato plant infested by 10 second‐instar *P. absoluta* larvae for 48 h (the larvae were removed prior to adding the test adults) was placed in the first jar. For the rest of the jars, one uninfested tomato plant was placed in each jar. Twenty 2‐day‐old mated *P. absoluta* female adults were released into jar 1. After 24 h, the distribution of female adults in the system was recorded. The dispersal experiment was conducted in a climate chamber (25 ± 1°C, 60% ± 5% RH, 16 h: 8 h L: D photoperiod), and each treatment was repeated 9 times (replicates).

### 4.3 Collection and Quantification of Headspace VOCs

Volatiles of uninfested and herbivore‐infested tomato plants were collected by a dynamic headspace collection system and analyzed by gas chromatography‐mass spectrometry (GC‐MS) [59]. An uninfested tomato plant, a *P. absoluta*‐infested tomato plant (10 second‐instar larvae infested for 48 h), an *S. exigua*‐infested tomato plant (5 second‐instar larvae infested for 48 h), or a *B. tabaci*‐infested tomato plant (20 nymphs infested for 48 h) was placed in a 5‐L glass jar. Air (500 mL·min^−1^) was pumped into the system and filtered through double‐distilled water, activated charcoal, and molecular sieves. Volatiles were trapped in a glass tube (10 cm long, 5 mm diameter) that contained 50 mg of 80/100 mesh Porapak‐Q (Altech Assoc.) under continuous light (20 000 ± 1 000 Lx) for 2 h in a climate chamber (25 ± 1°C, 60% ± 5% RH).

Porapak‐Q traps were each rinsed with 200 µL of dichloromethane containing 400 ng of nonyl acetate as an internal standard. Eluants were analyzed with a Shimadzu GC‐2010 plus GC‐MS (Shimadzu) equipped with a Rxi‐5MS (30 m–0.32 mm i.d., 0.25‐µm film thickness) column. Splitless injections (1 µL) into the inlet port (250°C) transferred the mixture onto the column with helium carrier gas at 1 mL/min and separated with the following temperature program: 40°C initial temperature for 3 min, which was increased at 6°C/min to 220°C and held for 5 min. The MS detector was operated in electron impact mode (70 eV), and mass spectra were recorded from 33 amu to 300 amu. Compounds were identified by comparing mass spectra with those of authentic standards or the NIST 22 library. Quantification of identified compounds was based on comparisons of their peak areas with the nonyl acetate internal standard. For each treatment, volatile collection was repeated 5 times, and collections were made for each treatment in parallel on each experimental day.

### 4.4 Identification of HIPVs That Trigger Dispersal of *P. absoluta* Females

Differentially accumulated volatile compounds between infested and *P. absoluta*‐infested tomato plants were identified based on the described quantification procedures. Electroantennographic (EAG) responses of *P. absoluta* females to 10 differentially accumulated volatile compounds were determined using an EAG detection system (Stimulus Air Controller CS‐55 and SYNTECH IDIC2; Syntech, Hilversum, the Netherlands). The pure compounds were diluted in a gradient with paraffin oil to four concentrations (0.1, 1, 10, and 100 µg/mL); 10 µL of each was applied to a piece of filter paper (5 mm × 2 cm), which was placed into a Pasteur pipette. For each test chemical, 10 µL of paraffin oil was used as a control. The stimulus was made by introducing the test volatile to the 2‐day‐old *P. absoluta* female antenna at a flow rate of 25 mL/min for 2 s and with an interval of 60 s for the next stimulus. The test order was paraffin oil, the test compound (concentrations from low to high), and paraffin oil. The test compound of each concentration was performed on three antennae. Relative EAG values were reported as the percentage of the response to paraffin oil [61].

The behavioral responses of *P. absoluta* females to volatile compounds with strong EAG responses [EAG response (% against paraffin) over 180] were tested using a Y‐tube olfactometer. Solutions of each compound were prepared in paraffin oil at a gradient of four concentrations (0.1 µg/mL, 1 µg/mL, 10 µg/mL, and 100 µg/mL), and 10 µL was pipetted onto a piece of clean filter paper (1 × 1 cm), which was then transferred to a glass flask as the test odor source. Filter paper with 10 µL of paraffin oil in a glass flask was used as a control odor source. Two‐day‐old *P. absoluta* females were used for olfactometer bioassays. The procedure was the same as described above. One hundred females were tested for each compound at each concentration.

The effects of two repellent compounds, (Z)‐3‐hexenol and benzaldehyde, on the dispersal of *P. absoluta* females were evaluated in the wind tunnel setup. 100 µg (Z)‐3‐hexenol or benzaldehyde was applied onto a piece of clean filter paper (1 × 1 cm) and attached to a tomato plant in the “adult release” jar. Tomato plants with 100 ng paraffin oil on filter paper were used as a control. The procedure of dispersal experiments for each treatment was the same as described above. Each treatment was repeated 9 times (replicates).

### 4.5 Identification of Infestation‐Induced Benzaldehyde‐Release‐Related Tomato Genes

Gene expression analyses of benzaldehyde biosynthesis‐ and stomatal‐related genes in response to *P. absoluta* larvae infestation were carried out based on our previously reported RNA‐seq data [46]. The benzaldehyde biosynthesis‐ and stomatal‐related genes were annotated with the Gene Ontology (GO) function. Expression differentiation analyses were conducted with the DESeq2 R package (v. 1.18.0)75. Genes with log2 (fold change) ≥ 1 and ≤‐1, and *p*‐value < 0.05 were considered as differentially expressed genes (DEGs). The expression of selected DEGs was validated by quantitative real‐time PCR (RT‐qPCR) analyses as previously described [46]. RT‐qPCR was conducted on a Bio‐RadCFX96 Touch Real‐time PCR Detection System instrument (Bio‐Rad, Hercules, CA, USA) using iTaq Universal SYBR Green Supermix (BIO‐RAD). The *β‐tubulin* (*TUB*) gene was used as the internal standard to normalize the variations in gene expression. The primers used are listed in Supplemental Table S4.

To examine the stomatal response to *P. absoluta* larvae infestation at the tissue level, the stomata of uninfested, *P. absoluta*‐infested, *B. tabaci*‐infested, and *S. exigua*‐infested tomato plants were observed. Approximately 1 cm^2^ of leaf tissue was excised from an infested leaf next to *P. absoluta* larvae feeding site using a razor blade. Each leaf section was then mounted on a slide and immediately observed using a Zeiss Axio Observer Z1 Microscope (Carl Zeiss, Jena, Germany) under 400× magnification. Five samples were prepared for each treatment, and three pictures were selected for stomata aperture quantification. In addition, the ultra‐microstructure of stomata on uninfested and *P. absoluta*‐infested tomato plants was observed by scanning electron microscopy (TM‐300, Hitachi, Japan) according to a previously described method [62]. The total volatile release of uninfested and *P. absoluta*‐infested tomato plants was collected as described above and calculated by summing all quantified volatiles.

### 4.6 Functional Analysis of IQM1 Gene Using VIGS Assays

Virus‐induced gene silencing (VIGS) was used to evaluate how knocking down *IQM1* gene expression influenced stomata opening, VOCs release, and *P. absoluta* performance. A 342‐bp fragment of the *IQM1* gene and A 435‐bp fragment of the *EGFP* gene were PCR‐amplified from cDNA of tomato leaves using specific primers (Table S5). The PCR products were cloned into the pTRV2 vector to construct pTRV2‐ IQM1 and pTRV2‐GFP, respectively (Figure 4A). The pTRV1, recombinant pTRV2‐IQM1, and pTRV2‐GFP were introduced into *Agrobacterium tumefaciens* GV3101, which was vacuum infiltrated into tomato seeds (Figure 4B) [63]. The infiltrated tomato seeds were planted in pots and kept in a growth chamber (25 ± 1°C, 60% ± 5% RH, 16 h: 8 h L: D photoperiod). When the plants reached the five‐fully‐expanded‐leaves stage (Figure S5), leaf RNA was extracted to synthesize cDNA [46]. The expression efficiency of the IQM1 or GFP genes was determined by RT‐qPCR (primers given in Table S4). The successfully infected tomato plants (VIQM1 tomato plants and VGFP tomato plants) were used for further study.

To assess the effect of *IQM1* silencing on *P. absoluta* response to larvae infestation, four types of tomato plants were prepared: (1) uninfested VGFP tomato plants; (2) infested VGFP tomato plants, where each plant was infested with 10 *P. absoluta* second instar larvae for 48 h, and the larvae were removed afterward; (3) uninfested VIQM1 tomato plants; (4) infested VIQM1 tomato plants, where each plant was infested with 10 *P. absoluta* second instar larvae for 48 h, and the larvae were removed afterward. Female oviposition choices were investigated with cage experiments, and Y‐tube olfactometers were used to compare female behavioral responses to pairs of contrasting plant treatments.

The dispersal of *P. absoluta* females on larvae‐infested VGFP and VIQM1 plants was evaluated in the wind tunnel setup described above.

### 4.7 Genomic Editing of IQM1 and Mutant Characterization

A CRISPR/Cas9 system optimized for tomato was used to edit IQM1 to generate a single‐gene knockout mutant *S. lycopersicum cv*. ZF202. Guide RNAs (gRNAs) were designed by the CRISPR‐P 2.0 web tool (http://crispr.hzau.edu.cn/cgi‐bin/CRISPR2/CRISPR), synthesized and cloned into a CRISPR/Cas9 vector, as described previously [64]. The constructed CRISPR/Cas9‐gRNA vector was transformed into *A. tumefaciens* (GV3101). The *A. tumefaciens* cells (GV3101) were cultured to an OD600 of 0.4 and used to infect cotyledon explants from one‐week‐old healthy tomato seedlings. After co‐cultivation, explants were cultivated on shoot induction medium, shoot elongation medium, and rooting medium as previously described [65]. Finally, well‐developed plantlets with roots were planted into pots and maintained in a climate chamber (25 ± 1°C, 60% ± 5% RH, 16 h: 8 h L: D photoperiod). Knockout mutants were identified by DNA sequencing. The primers for vector construction and mutation detection are listed in Table S6. The confirmed mutant plants were selfed, and homozygous IQM1 mutant plants from the F1 generation were used for further analysis (Figure S6).

To assess the effect of *IQM1* knocking‐out on the herbivory‐induced VOCs release, VOCs were collected from the four treatment groups: (1) uninfested wild‐type tomato plants; (2) infested wild‐type tomato plants (each plant was infested with 10 *P. absoluta* second instar larvae for 48 h, and the larvae were removed afterward); (3) uninfested IQM1 mutants; (4) infested IQM1 mutants (each plant was infested with 10 *P. absoluta* second instar larvae for 48 h, and the larvae were removed afterward). VOCs from 7 to 9 replicate plants from each treatment group were collected and analyzed as described above. The stomata of uninfested, *P. absoluta*‐infested, *B. tabaci*‐infested, and *S. exigua*‐infested MT2‐1 tomato leaves were observed as described above.

### 4.8 Large‐Scale Glasshouse Assays of *P. absoluta* Dispersal and Population Growth

To assess the effect of *IQM1* abrogation on *P. absoluta*’s performance as an invasive pest, adult dispersal and population growth were investigated in a thrice‐replicated large‐scale (3.5 m×3.5 m×2 m) glasshouse experiment, which had two spatially separated plant populations: wild‐type and IQM1 mutant tomato plants. Each population contained 144 potted tomato plants placed in 12 rows ×12 columns. In the center of each population, a single tomato plant was infested with 10 *P. absoluta* second‐instar larvae for 48 h, and the larvae were removed. Then five male‐female pairs of newly emerged *P. absoluta* adults were released onto the infested plant in the middle of the cage and allowed to disperse and oviposit. The number of F_1_ eggs laid by the F_0_ generation (five male‐female pairs) was recorded every day, and the total number was calculated. When the F_1_ adults emerged, the number of F_2_ eggs laid by F_1_ adults was also recorded every day, and the total number was calculated. Meanwhile, 100 F_2_ eggs and 100 newly hatched larvae were randomly selected on different tomato plants, and the following parameters were measured daily: egg hatchability, larval survivorship, and pupation rate. To determine the eclosion rate and sex ratio, 50 pupae were randomly selected and covered with net cages (12 cm × 12 cm× 12 cm); the eclosion rate and the proportion of female adults were determined. Newly emerged females were paired with males in cages, and the number of laid eggs was recorded for each female.

The population trend index (*I*) was calculated based on the Morris‐Watt model with some modification described as: *I* = *N*_1_ / *N*_0_ = *S_E_* × *S_L_* × *S_P_* × *S_A_* × *F* × *P_F_* × *P_♀_* [66], where *N*_1_ and *N*_0_ are the numbers of next generation and current generation, respectively; *S_E_*, *S_L_*, *S_P_*, and *S_A_* are the respective egg hatching rate, larval survival rate, pupation rate, and eclosion rate; *F* is the number of initial eggs; *P_F_* is the average number of eggs laid per female; and *P_♀_* is the proportion of female adults. For the F_1_ generation, the Population Trend Index (*I*) was calculated directly as the ratio of the total number of eggs laid by F_1_ adults to the total number of eggs laid by F_0_ adults. For the F_2_ generation, the Population Trend Index (*I*) was calculated as *I* = *S_E_* × *S_L_* × *S_P_* × *S_A_* × *F* × *P_F_* × *P_♀_*.

To calculate the indices of patchiness (Kuno's *C*_A_), the number of *P. absoluta* larvae per tomato plant was recorded every day, and the peak number of larvae was used. Kuno's CA was calculated as follow*s*: $CA=1/K=\left(S^{2}-\bar{X}\right)/{\bar{X}}^{2}$, where *k* is a non‐negative characteristic exponent, *S* ^2^ is the mean of sample variance, $\bar{X}$ is the mean of the population. When *C_A_* = 1, the population distribution is random; when *C_A_* > 1, the population distribution is patchy; when *C_A_* < 1, the population distribution is uniform [67].

### 4.9 Statistical Analyses

Statistical analyses were conducted using SPSS 22.0 (IBM SPSS, Somers, NY, USA). The data distributions were evaluated using nonparametric Kolmogorov‐Smirnov tests (*p* < 0.05). For Y‐tube olfactometer behavioral assays, data were analyzed using chi‐square tests. For oviposition choice and wind tunnel dispersal assays, count data following Poisson distributions were analyzed using Generalized Linear Mixed Model (GLMM) analysis based on Poisson or binomial distributions followed by LSD or likelihood ratio tests. For larval survival curves, the Kaplan‐Meier survival curves of *P. absoluta* in two treatments were compared using a Log‐Rank test. For EAG responses, stomatal aperture, gene expression, life table parameters, and quantitative volatile comparisons, normally distributed data with two treatments were analyzed using Student's *t*‐test (*p* < 0.05) and with more than two treatments were analyzed using one‐way ANOVA followed by pairwise comparisons using Tukey's HSD analysis (*p* < 0.05).

For the analysis of volatiles, two independent collection experiments were conducted. In the first experiment (Table S1), volatiles from four treatments (uninfested, *P. absoluta*‐, *B. tabaci*‐, and *S. exigua*‐infested plants) were compared with five replicates per treatment. Differences among treatments were assessed by one‐way ANOVA with Tukey's HSD, and OPLS‐DA was performed to visualize overall separation among treatment groups. In the second experiment (Tables S2 and S3), we focused specifically on pairwise comparisons between uninfested and *P. absoluta*‐infested plants (wild‐type and IQM1 mutant, respectively) with increased replication (*n* = 9). For these two‐group analyses, differential metabolites were determined by VIP (VIP ≥ 1) and absolute Log2FC (|Log2FC| ≥ 1.0). VIP values were extracted from the OPLS‐DA results, which contained the score and permutation plots, and were generated using the R package MetaboAnalystR. The data was log transform (log2) and mean‐centered before OPLS‐DA. To avoid overfitting, a permutation test (200 permutations) was performed.

## Author Contributions

X.L. and Y.L. designed and supervised the projects. L.C., Y.W., S.Z. and F.U. performed the experiments. L.C. and W.H. analyzed the data and created the figures. L.C., X.L., I.T.B. and Y.L. wrote the paper. L.D., H.J.B. and Y.H. provided technical support and revised the manuscript. All the authors have read and approved the final version of the manuscript. All the listed authors have read and approved the final version, including all details and images.

## Funding

This work was supported by the National Key R&D Program of China (2023YFD1401200), the Joint Funds of the National Natural Science Foundation of China (U22A20489), the Major Science and Technology Projects in Xinjiang (2023A02006), the National Natural Science Foundation of China (32272524), and the Zhejiang High‐level Talents Special Support Program (2023R5249).

## Conflicts of Interest

The authors declare no conflict of interest.

## Supporting information




**Supporting File**: advs77207‐sup‐0001‐SuppMat.docx.

## Data Availability

The data that support the findings of this study are available from the corresponding author upon reasonable request.
