# Supplementary material for: Herbivory‐Induced Stomatal Opening Triggers the Dispersal of an Invasive Insect Herbivore
Source: Adv Sci (Weinh). 2026 Aug 17;13(62):e77207. doi: 10.1002/advs.77207 (PMC13479857; doi:10.1002/advs.77207)
Supplement: Supplementary file 1 [file ADVS-13-e77207-s001.docx]

Supporting Information

Herbivory induced stomatal opening triggers the dispersal of an invasive insect herbivore

Limin Chen, Xiaowei Li, Yaru Wang, Farman Ullah, Shuxing Zhou, Lemeng Dong, Weizhong He, Harro J. Bouwmeester, Youming Hou,* Ian T. Baldwin,* Yaobin Lu*

**Figures S1-8, Tables S1-7**

| **Figures and Tables** | **Content** | **Page** |
| --- | --- | --- |
| Figure S1 | Larval infestation influences *Phthorimaea absoluta* pupation and oviposition behavior and the oviposition behavior of *Phthorimaea absoluta*, *Spodoptera exigua* and *Bemisia tabaci* adults | 2 |
| Figure S2 | Electroantennogram (EAG) responses of *Phthorimaea absoluta* to HIPVs | 4 |
| Figure S3 | Heatmap of the effect of *Phthorimaea absoluta* larval infestations on the expression of benzaldehyde biosynthesis-related and stomatal-related genes in tomato | 5 |
| Figure S4 | The effect of *B. tabaci* and *S. exigu*a infestation on stomatal apertures of tomato leaves, and transcript levels of *IQM1* gene. | 6 |
| Figure S5 | Effect of IQM1 silencing on tomato growth | 7 |
| Figure S6 | Effect of IQM1 knockout on tomato growth. | 8 |
| Figure S7 | The effect of *P. absoluta*, *B. tabaci* and *S. exigua* infestation on stomatal apertures of MT2-1 tomato leaves. | 10 |
| Figure S8 | Plant volatiles emitted by uninfested and P. absoluta-infested wildtype (WT) and mutant line (MT2-1) tomato plants | 11 |
| Figure S9 | Dispersal and population growth experiment | 12 |
| Figure S10 | Population aggregation patterns of *P. absoluta* in F2 generation | 13 |
| Figure S11 | The infestation situation of late stage of field-simulation experiments in the glasshouse | 14 |
| Table S1 | Plant volatiles emitted by uninfested, *P. absoluta*-infested, B. tabaci-infested, and S. exigua-infested tomato plants | 15 |
| Table S2 | Plant volatiles emitted by uninfested and *P. absoluta*-infested tomato plants | 17 |
| Table S3 | Plant volatiles emitted by MT2-1 uninfested and larval infested tomato plants | 19 |
| Table S4 | qRT-PCR primers of 8 selected genes in tomato | 21 |
| Table S5 | Primers used for VIGS | 22 |
| Table S6 | Primers used for DNA constructs | 23 |
| Table S7 | Life table parameters of of Phthorimaea absoluta on wild type and IQM1 mutant tomato plants | 24 |

**Figures**

**Figure S1.** Larval infestation influences *Phthorimaea absoluta* pupation and oviposition behavior and the oviposition behavior of *Phthorimaea absoluta,* *Spodoptera exigua* and *Bemisia tabaci* adults. (A) The pupation rate of *P. absoluta* larvae fed on uninfested and larvae-infested tomato plants (n = 20 replicates with 5 larvae per replicate). (B), (D), (E). The number of eggs laid by *P. absoluta* females ((B), n = 20 adults/cage), *B. tabaci* females ((D), n = 40 adults/cage), and *S. exigua* females (E, n = 10 adults/cage) on uninfested and *P. absoluta* larvae-infested (n = 10 larvae/plant) tomato plants in choice tests in cages (n = 11 cages). (C) Proportion of *P. absoluta* females choosing different odor sources in Y-tube olfactometers (n = 100 adults). (F), (G), (H) The number of eggs laid by *P. absoluta* females ((F), n = 20 adults/cage), *B. tabaci* females ((G), n = 40 adults/cage), and *S. exigua* females ((H), n = 10 adults/cage) on uninfested and *B. tabaci* nymph-infested (n = 20 nymphs/plant) tomato plants in choice tests in cages (n = 11 cages). (I), (J), (K). The number of eggs laid by *P. absoluta* females ((I), n = 20 adults/cage), *B. tabaci* females ((J), n = 40 adults/cage), and *S. exigua* females ((K), n = 10 adults/cage) on uninfested and *S. exigua* larvae-infested (n = 5 larvae /plant) tomato plants in choice tests in cages (n = 11 cages). Differences between means (±SE) in (A) was analyzed using two-tailed unpaired *t*-test. The count data following Poisson distributions in (B), (D)-(K) were analyzed using General Linear Model (GLMM) analysis based on Poisson distributions followed by LSD multiple range tests. Y-tube behavioral choice data in (C) were analyzed using chi-square tests. Asterisks indicate significant differences (**P* < 0.05, ***P* < 0.01, ****P* < 0.001). For choice data, exact *P*-values = 0.942 (4 h), 0.831 (8 h), 0.888 (12 h), 0.395 (18 h), 0.029 (24 h), 0.01 (36 h), 0.042 (48 h), 0.037 (60 h), and 0.108 (72 h). For oviposition data, exact *P*-values < 0.001 (B), = 0.121 (D), = 0.902 (E), = 0.001 (F), = 0.029 (G), = 0.001 (H), = 0.016 (I), = 0.002 (J), = 0.003 (K).

**Figure S2.** Electroantennogram (EAG) responses of *Phthorimaea absoluta* to HIPVs. Bar graphs show the mean ± SE from 5 replicates. Different lowercase letters indicate significant differences (one-way ANOVA, Tukey’s HSD test, *P* < 0.05). β-pinene (*F*_1,8_ = 2.985, *P* = 0.062), methyl salicylate (*F*_1,8_ = 2.231, *P* = 0.112), hexanal (*F*_1,8_ = 9.747, *P* = 0.001), (Z)-3-hexenol (*F*_1,8_ = 10.540, *P* < 0.001), α-pinene (*F*_1,8_ = 3.926, *P* = 0.028), benzaldehyde (*F*_1,8_ = 3.169, *P* = 0.050), β-pinene (*F*_1,8_ = 2.95, *P* = 0.062), 2-carene (*F*_1,8_ = 16.806, *P* < 0.001), α-phellandrene (*F*_1,8_ = 19.693, *P* < 0.001), α-terpinene (*F*_1,8_ = 13.202, *P* < 0.001), γ-terpinene (*F*_1,8_ = 27.625, *P* < 0.001), terpinolene (*F*_1,8_ = 21.521, *P* < 0.001), methyl salicylate (*F*_1,8_ = 2.340, *P* = 0.112), α-cedrene (*F*_1,8_ = 7.450, *P* = 0.002), (E)-β-caryophyllene (*F*_1,8_ = 5.126, *P* = 0.011), γ-elemene (*F*_1,8_ = 14.569, *P* < 0.001), α-caryophyllene (*F*_1,8_ = 8.539, *P* = 0.001). EAG response (% against paraffin) over 180 represents a “strong” EAG response.

**Figure S3.** Heatmap of the effect of *Phthorimaea absoluta* larval infestations on the expression of benzaldehyde biosynthesis-related and stomatal-related genes in tomato. Log2-transformed RPKM values are plotted. (A) 16 benzaldehyde biosynthesis-related genes. (B)-(E) 140 stomatal-related genes. Transcriptome data were obtained from the uninfested and infested (with one 2^nd^ instar *P. absoluta* larva for 48 h, before larval removal) third nodal leaves (without veins) of tomato plants with five fully expanded leaves. Each sample consisted of 1 g of leaves; 3 samples were collected for each treatment. Leaves were harvest at 48 h after infestation.

**Figure S4.** The effect of *B. tabaci and S. exigua* infestation on stomatal apertures and transcript levels of *IQM1* gene of tomato leaves. (A) Representative images of leaf stomata of uninfested, *B. tabaci*-infested and *S. exigua*-infested tomato plants from light microscopy (bars 20 μm). (B) The stomatal aperture of tomato leaves from uninfested and *B. tabaci*-infested tomato plants (*t* = 0.345, *df* = 98, *P* = 0.731, n = 50 stomata). (C) The stomatal aperture of tomato leaves from uninfested and *S. exigua*-infested tomato plants (*t* = 5.519, *df* = 98, *P* < 0.0001, n = 50 stomata). (D) Transcript levels of *IQM1* gene in uninfested and *B. tabaci*-infested tomato plants (*t* = 1.471, *df* = 8, *P* = 0.179, n = 5 biological replicates). (E) Transcript levels of *IQM1* gene in uninfested and *S. exigua* -infested tomato plants (*t* = 0.269, *df* = 8, *P* = 0.795, n = 5 biological replicates). Differences between means (±SE) in (B-E) were analyzed using two-tailed unpaired *t*-test. Asterisks indicate significant differences (**P* < 0.05, ***P* < 0.01, ****P* < 0.001).

**Figure S5.** Effect of *IQM1* silencing on tomato growth. (A) Image of tomatoes 30 days post-VIGS treatment. *IQM1* silencing and GFP-expression effects on plant height, stem diameter, and chlorophyll content of plants 30 (B) and (C) 40 days post-VIGS treatment. Exact *P*-values for 30-day data: plant height (*t* = 0.727, *df* = 40, *P* = 0.727), stem diameter (*t* = 0.633, *df* = 40, *P* = 0.883), and chlorophyll content (*t* = 0.449, *df* = 40, *P* = 0.901). Exact *P*-values for 40-day data: plant height (*t* = 2.100, *df* = 40, *P* = 0.470), stem diameter (*t* = 1.303, *df* = 40, *P* = 0.051), and chlorophyll content (*t* = 0.801, *df* = 40, *P* = 0.264). Differences between means (±SE) in (B) and (C) were analyzed using two-tailed unpaired *t*-test. NS indicates no significant differences.

**Figure S6.** Effect of *IQM1* knockout on tomato growth. (A) Image of WT and mt2-1 45-day-old plants. Plant height, stem diameter, and chlorophyll content of WT and MT2-1 tomato plants at 25 (B), 35 (C) and 45 (D) -day-old. Exact *P*-values for 25-day data: plant height (*t* = 0.215, *df* = 40, *P* = 0.831), stem diameter (*t* = 1.255, *df* = 40, *P* = 0.217), and chlorophyll content (*t* = 1.189, *df* = 40, *P* = 0.242). Exact *P*-values for 35-day data: plant height (*t* = -0.372, *df* = 40, *P* = 0.712), stem diameter (*t* = 0.569, *df* = 40, *P* = 0.572), and chlorophyll content (*t* = -1.197, *df* = 40, *P* = 0.238). Exact *P*-values for 45-day data: plant height (*t* = 0.901, *df* = 40, *P* = 0.373), stem diameter (*t* = 1.020, *df* = 40, *P* = 0.314), and chlorophyll content (*t* = -1.474, *df* = 40, *P* = 0.148). Differences between means (±SE) in (B) and (C) were analyzed using two-tailed unpaired *t*-test. NS indicates no significant differences.

**Figure S7.** The effect of *P. absoluta*, *B. tabaci and S. exigua* infestation on stomatal apertures of MT2-1 tomato leaves. (A) Representative images of leaf stomata of uninfested, *P. absoluta*-infested, *B. tabaci*-infested and *S. exigua*-infested tomato plants from light microscopy (bars 20 μm). (B) The stomatal aperture of tomato leaves from uninfested, *P. absoluta*, *B. tabaci and S. exigua* infestation tomato plants (*F*_3,196_ = 7.202, *P* = 0.0001, n = 50 stomata). Differences among means (±SE) in (B) were analyzed using one-way ANOVA followed by Tukey’s HSD test analysis. Different letters indicate significant differences among treatments.

 **Figure S8.** Plant volatiles emitted by uninfested and *P. absoluta*-infested wildtype (WT) and mutant line (MT2-1) tomato plants (n = 9 plants). 1. hexanal; 2. (Z)-3-hexenol; 3. p-xylene; 4. α-pinene; 5. benzaldehyde; 6. 3,7,7-trimethyl-1,3,5-cycloheptatriene; 7. β-pinene; 8. β-myrcene; 9. 2-carene; 10. α-phellandrene; 11. α-terpinene; 12. p-cymene; 13. β-phellandrene; 14. benzeneacetaldehyde; 15. (Z)-β-ocimene; 16. γ-terpinene; 17. terpinolene; 18. linalool; 19. nonanal; 20. (Z)-3-hexenyl butanoate; 21. dill ether; 22. methyl salicylate; 23. decanal; 24. p-cumenol; 25. p-cymen-7-ol; 26. δ-elemene; 27. β-elemene; 28. α-cedrene; 29. (E)-β-caryophyllene; 30. γ-elemene; 31. 6,9-guaiadiene; 32. α-caryophyllene; 33. trans-farnesol; 34. TMTT; 35. diethyl phthalate.

**Figure S9.** Dispersal and population growth experiment. Early (A) (day= 1) and late (B) (day= 65) stage of the experiment.

 **Figure S10.** Population aggregation patterns of *P. absoluta* in F2 generation on wild-type tomato plants and IQM1 mutant line in replicates 2 and 3.

**Figure S11.** The infestation situation of late stage of field-simulation experiments in the glasshouse with excessive infestation and mortality of *Phthorimaea absoluta* larvae in populations of mutant plants (right), but not in populations of WT plants (left).

**Tables**

**Table S1.** Plant volatiles emitted by uninfested, *P. absoluta*-infested, *B. tabaci*-infested, and *S. exigua*-infested tomato plants (ng⋅h^-1^plant^-1^).

| Peak No. | RT | Compound | Formula | CAS | Chemical class | Uninfested plants | *P. absoluta* infested | *B. tabaci* infected | *S. exigua* infested | *F*_3,16_ | *P* |
| --- | --- | --- | --- | --- | --- | --- | --- | --- | --- | --- | --- |
| 1 | 5.644 | hexanal | C6H14 | 66-25-1 | Aldehyde | 0.11±0.038a | 0.28±0.116a | 0.24±0.119a | 0.13±0.038a | 2.53 | 0.0939 |
| 2 | 7.164 | (Z)-3-hexenol | C6H12O | 928-96-1 | Alcohol | 0±0b | 2.87±0.82a | 1.8±0.751ab | 0.8±0.179ab | 12.204 | 0.0002 |
| 3 | 7.568 | p-xylene | C8H10 | 106-42-3 | Aromatic compound | 0.71±0.209a | 1.03±0.116a | 1.21±0.353a | 0.57±0.172a | 5.59 | 0.0081 |
| 4 | 9.392 | α-pinene | C10H16 | 80-56-8 | Monoterpene | 2.95±0.515a | 3.36±0.314a | 2.3±0.077a | 2.61±0.046a | 6.536 | 0.0043 |
| 5 | 10.19 | benzaldehyde | C10H14 | 202-860-4 | Aldehyde | 0±0a | 0.91±0.09b | 0±0a | 0±0a | 46.415 | 4.05E-08 |
| 6 | 10.48 | 3,7,7-trimethyl-1,3,5-cycloheptatriene | C10H14 | 3479-89-8 | Homoterpene | 2.45±0.245a | 3.92±0.218a | 3.28±0.68a | 2.54±0.119a | 15.385 | 5.65E-05 |
| 7 | 10.66 | β-pinene | C10H16 | 18172-67-3 | Monoterpene | 0.09±0.023a | 0.33±0.048b | 0.1±0.043a | 0.13±0.032a | 1.471 | 0.26 |
| 8 | 11.01 | β-myrcene | C10H16 | 123-35-3 | Monoterpene | 0.67±0.029b | 1.15±0.031a | 1.14±0.087a | 0.67±0.023b | 1.956 | 0.1614 |
| 9 | 11.29 | 2-carene | C10H16 | 4497-92-1 | Monoterpene | 18.41±1.995a | 21.22±0.671a | 16.98±0.799a | 18.03±0.692a | 5.27 | 0.0102 |
| 10 | 11.48 | α-phellandrene | C10H16 | 99-83-2 | Monoterpene | 3.03±0.222b | 4.42±0.179a | 3.2±0.363b | 3.19±0.292b | 1.326 | 0.3007 |
| 11 | 11.78 | α-terpinene | C10H18O | 99-86-5 | Monoterpene | 0.35±0.027b | 0.37±0.076ab | 0.44±0.019ab | 0.56±0.049a | 5.503 | 0.0086 |
| 12 | 12 | p-cymene | C10H14 | 99-87-6 | Monoterpene | 0.98±0.076b | 1.47±0.013a | 0.92±0.156b | 0.61±0.068b | 7.56 | 0.0023 |
| 13 | 12.2 | β-phellandrene | C10H16 | 99-86-5 | Monoterpene | 57.55±5.043a | 55.51±1.908a | 50.5±4.203a | 48.46±1.57a | 3.138 | 0.0545 |
| 14 | 12.49 | benzeneacetaldehyde | C10H12O | 144164-15-8 | Aldehyde | 0.09±0.02c | 0.38±0.017a | 0.27±0.069ab | 0.12±0.014bc | 19.963 | 1.17E-05 |
| 15 | 12.59 | (Z)-β-ocimene | C10H16 | 13877-91-3 | Monoterpene | 0.54±0.041b | 1.33±0.054a | 0.89±0.142b | 0.72±0.096b | 4.302 | 0.0209 |
| 16 | 12.93 | γ-terpinene | C10H16 | 99-85-4 | Monoterpene | 0.79±0.198b | 1.62±0.142a | 1.48±0.122a | 0.65±0.105b | 0.334 | 0.8007 |
| 17 | 13.67 | terpinolene | C10H16 | 586-62-9 | Monoterpene | 0.69±0.084ab | 1.01±0.065a | 0.73±0.152ab | 0.46±0.009b | 11.385 | 0.0003 |
| 18 | 13.97 | linalool | C10H18O | 78-70-6 | Monoterpene | 0.85±0.24b | 1.41±0.151ab | 1.69±0.21a | 0.79±0.071b | 5.835 | 0.0068 |
| 19 | 14.15 | nonanal | C9H18O | 124-19-6 | Aldehyde | 0.38±0.108b | 0.66±0.095ab | 0.76±0.097a | 0.34±0.014b | 5.856 | 0.0067 |
| 20 | 16.05 | (Z)-3-hexenyl butanoate | C10H18O2 | 16491-36-4 | Ester | 0.07±0.006c | 0.17±0.018a | 0.14±0.019ab | 0.09±0.005bc | 4.615 | 0.0165 |
| 21 | 16.29 | dill ether | C10H16O | 70786-44-6 | Monoterpene | 0.22±0.053a | 0.51±0.116a | 0.45±0.09a | 0.26±0.03a | 4.749 | 0.0149 |
| 22 | 16.43 | methyl salicylate | C8H8O3 | 119-36-8 | Benzenoid ester | 0.19±0.098c | 1.68±0.13a | 0.81±0.184b | 0.46±0.075bc | 2.04 | 0.1488 |
| 23 | 16.74 | decanal | C10H20O | 112-31-2 | Aldehyde | 0.22±0.07ab | 0.41±0.08a | 0.38±0.028ab | 0.17±0.013b | 4.676 | 0.0157 |
| 24 | 17.15 | p-cumenol | C9H12O | 99-89-8 | Alkylphenol | 0.12±0.011b | 0.31±0.061a | 0.22±0.041ab | 0.12±0.008b | 6.108 | 0.0057 |
| 25 | 17.64 | p-cymen-7-ol | C10H14O | 536-60-7 | Monoterpenes | 0.84±0.186a | 1.05±0.048a | 0.82±0.047a | 0.39±0.015b | 4.927 | 0.013 |
| 26 | 18.09 | δ-elemene | C15H24 | 20307-84-0 | Sesquiterpene | 0.16±0.03ab | 0.21±0.037a | 0.2±0.011ab | 0.1±0.014b | 5.858 | 0.0067 |
| 27 | 18.6 | β-elemene | C15H24 | 515-13-9 | Sesquiterpene | 0.26±0.039b | 0.39±0.092ab | 0.54±0.06a | 0.18±0.014b | 9.422 | 0.0008 |
| 28 | 21.75 | α-cedrene | C15H24 | 469-61-4 | Sesquiterpene | 0.2±0.083b | 0.33±0.056ab | 0.51±0.093a | 0.19±0.007b | 5.271 | 0.0102 |
| 29 | 21.82 | (E)-β-caryophyllene | C15H24 | 87-44-5 | Sesquiterpene | 0.64±0.216b | 1.88±0.089a | 2.1±0.226a | 0.99±0.083b | 1.325 | 0.3009 |
| 30 | 21.94 | γ-elemene | C15H24 | 29873-99-2 | Sesquiterpene | 0.08±0.025b | 0.14±0.021ab | 0.19±0.033a | 0.07±0.003b | 4.047 | 0.0256 |
| 31 | 22.25 | 6,9-guaiadiene | C15H24 |  | Sesquiterpene | 0.08±0.02a | 0.2±0.033a | 0.16±0.046a | 0.07±0.021a | 1.304 | 0.3074 |
| 32 | 22.58 | α-caryophyllene | C15H24 | 6753-98-6 | Sesquiterpene | 0.19±0.074b | 0.57±0.048a | 0.61±0.079a | 0.26±0.019b | 2.559 | 0.0915 |
| 33 | 24.5 | trans-farnesol | C15H26O | 106-28-5 | Sesquiterpene | 0.26±0.077b | 0.52±0.076ab | 0.73±0.204a | 0.25±0.006b | 3.558 | 0.0382 |
| 34 | 24.88 | TMTT | C16H26 | 62235-06-7 | Terpenoid | 4.74±2.266b | 9.22±0.782ab | 11.82±2.453a | 5.6±0.534ab | 3.903 | 0.0287 |
| 35 | 25.11 | diethyl phthalate | C12H14O4 | 84-66-2 | Ester | 0.32±0.249a | 2.68±0.35a | 2.77±1.165a | 1.38±0.027a | 3.388 | 0.044 |

Note: Differences among the means (±SE) were analyzed using one-way ANOVA followed by Tukey’s HSD test analysis. Different letters indicate significant differences among treatments.

**Table S2.** Plant volatiles emitted by uninfested and *P. absoluta*-infested tomato plants (ng⋅h^-1^plant^-1^).

| Peak No. | RT | Compound | Formula | CAS | Chemical class | Uninfested plants | *P. absoluta* infested | VIP | *P*-value | Fold_Change | Type |
| --- | --- | --- | --- | --- | --- | --- | --- | --- | --- | --- | --- |
| 1 | 5.644 | hexanal | C6H14 | 66-25-1 | Aldehyde | 0.29±0.065 | 0.71±0.065 | 1.443 | 0.000 | 2.493 | up |
| 2 | 7.164 | (Z)-3-hexenol | C6H12O | 928-96-1 | Alcohol | 0±0 | 0.80±0.083 | 1.792 | 0.000 | Inf | up |
| 3 | 7.568 | p-xylene | C8H10 | 106-42-3 | Aromatic compound | 0.98±0.294 | 0.81±0.329 | 0.189 | 0.701 | 0.823 | insig |
| 4 | 9.392 | α-pinene | C10H16 | 80-56-8 | Monoterpene | 4.56±0.62 | 7.94±1.027 | 1.118 | 0.014 | 1.741 | up |
| 5 | 10.19 | benzaldehyde | C10H14 | 202-860-4 | Aldehyde | 0±0 | 0.56±0.21 | 1.058 | 0.028 | Inf | up |
| 6 | 10.48 | 3,7,7-trimethyl-1,3,5-cycloheptatriene | C10H14 | 3479-89-8 | Homoterpene | 3.10±0.497 | 5.01±0.87 | 0.870 | 0.078 | 1.620 | insig |
| 7 | 10.66 | β-pinene | C10H16 | 18172-67-3 | Monoterpene | 0.09±0.008 | 0.19±0.036 | 1.097 | 0.023 | 2.158 | up |
| 8 | 11.01 | β-myrcene | C10H16 | 123-35-3 | Monoterpene | 1.67±0.148 | 1.53±0.167 | 0.309 | 0.542 | 0.917 | insig |
| 9 | 11.29 | 2-carene | C10H16 | 4497-92-1 | Monoterpene | 33.04±4.228 | 50.55±5.431 | 1.037 | 0.022 | 1.530 | up |
| 10 | 11.48 | α-phellandrene | C10H16 | 99-83-2 | Monoterpene | 3.77±0.678 | 7.58±1.146 | 1.124 | 0.013 | 2.008 | up |
| 11 | 11.78 | α-terpinene | C10H18O | 99-86-5 | Monoterpene | 0.64±0.173 | 1.53±0.264 | 1.102 | 0.014 | 2.389 | up |
| 12 | 12 | p-cymene | C10H14 | 99-87-6 | Monoterpene | 0.64±0.056 | 0.85±0.111 | 0.734 | 0.129 | 1.316 | insig |
| 13 | 12.2 | β-phellandrene | C10H16 | 99-86-5 | Monoterpene | 74.91±7.459 | 106.62±6.433 | 1.222 | 0.005 | 1.423 | insig |
| 14 | 12.49 | benzeneacetaldehyde | C10H12O | 144164-15-8 | Aldehyde | 0.05±0.007 | 0.07±0.007 | 0.883 | 0.057 | 1.385 | insig |
| 15 | 12.59 | (Z)-β-ocimene | C10H16 | 13877-91-3 | Monoterpene | 0.59±0.102 | 0.86±0.106 | 0.801 | 0.086 | 1.457 | insig |
| 16 | 12.93 | γ-terpinene | C10H16 | 99-85-4 | Monoterpene | 0.34±0.039 | 0.53±0.061 | 1.050 | 0.022 | 1.548 | up |
| 17 | 13.67 | terpinolene | C10H16 | 586-62-9 | Monoterpene | 0.37±0.058 | 0.67±0.093 | 1.093 | 0.016 | 1.826 | up |
| 18 | 13.97 | linalool | C10H18O | 78-70-6 | Monoterpene | 0.3±0.017 | 0.18±0.015 | 1.551 | 0.000 | 0.591 | down |
| 19 | 14.15 | nonanal | C9H18O | 124-19-6 | Aldehyde | 0.14±0.015 | 0.17±0.014 | 0.491 | 0.281 | 1.161 | insig |
| 20 | 16.05 | (Z)-3-hexenyl butanoate | C10H18O2 | 16491-36-4 | Ester | 0.13±0.018 | 0.21±0.037 | 0.820 | 0.091 | 1.584 | insig |
| 21 | 16.29 | dill ether | C10H16O | 70786-44-6 | Monoterpene | 0.21±0.024 | 0.25±0.039 | 0.451 | 0.374 | 1.200 | insig |
| 22 | 16.43 | methyl salicylate | C8H8O3 | 119-36-8 | Benzenoid ester | 0.1±0.016 | 0.19±0.031 | 1.021 | 0.030 | 1.824 | up |
| 23 | 16.74 | decanal | C10H20O | 112-31-2 | Aldehyde | 0.11±0.012 | 0.13±0.015 | 0.343 | 0.493 | 1.118 | insig |
| 24 | 17.15 | p-cumenol | C9H12O | 99-89-8 | Alkylphenol | 0.16±0.014 | 0.19±0.015 | 0.576 | 0.212 | 1.165 | insig |
| 25 | 17.64 | p-cymen-7-ol | C10H14O | 536-60-7 | Monoterpenes | 0.11±0.021 | 0.14±0.023 | 0.590 | 0.263 | 1.341 | insig |
| 26 | 18.09 | δ-elemene | C15H24 | 20307-84-0 | Sesquiterpene | 0.16±0.055 | 0.16±0.034 | 0.040 | 0.974 | 0.987 | insig |
| 27 | 18.6 | β-elemene | C15H24 | 515-13-9 | Sesquiterpene | 0.24±0.073 | 0.23±0.048 | 0.030 | 0.966 | 0.984 | insig |
| 28 | 21.75 | α-cedrene | C15H24 | 469-61-4 | Sesquiterpene | 0.22±0.02 | 0.11±0.008 | 1.592 | 0.000 | 0.476 | down |
| 29 | 21.82 | (E)-β-caryophyllene | C15H24 | 87-44-5 | Sesquiterpene | 5.23±0.625 | 2.63±0.224 | 1.397 | 0.003 | 0.502 | down |
| 30 | 21.94 | γ-elemene | C15H24 | 29873-99-2 | Sesquiterpene | 0.12±0.009 | 0.06±0.006 | 1.543 | 0.000 | 0.523 | down |
| 31 | 22.25 | 6,9-guaiadiene | C15H24 |  | Sesquiterpene | 0.15±0.015 | 0.18±0.019 | 0.675 | 0.214 | 1.207 | insig |
| 32 | 22.58 | α-caryophyllene | C15H24 | 6753-98-6 | Sesquiterpene | 0.93±0.112 | 0.47±0.033 | 1.396 | 0.003 | 0.506 | down |
| 33 | 24.5 | trans-farnesol | C15H26O | 106-28-5 | Sesquiterpene | 0.02±0.001 | 0.03±0.003 | 0.953 | 0.040 | 1.447 | insig |
| 34 | 24.88 | TMTT | C16H26 | 62235-06-7 | Terpenoid | 2.44±0.334 | 2.99±0.591 | 0.331 | 0.440 | 1.221 | insig |
| 35 | 25.11 | diethyl phthalate | C12H14O4 | 84-66-2 | Ester | 0.62±0.079 | 0.79±0.14 | 0.531 | 0.312 | 1.274 | insig |

Note: Following discriminant analysis of orthogonal partial least squares with threshold of *P*-value < 0.05, Fold_change ≥ 1.5 and variable importance in projection (VIP) ≥ 1. Show the mean ± SE from 9 replicates.

**Table S3.** Plant volatiles emitted by MT2-1 uninfested and larval infested tomato plants (ng⋅h^-1^plant^-1^).

| Peak No. | RT | Compound | Formula | CAS | Chemical class | MT2-1 Uninfested plants | MT2-1 *P. absoluta* infested | VIP | *P*-value | Fold_Change | Type |
| --- | --- | --- | --- | --- | --- | --- | --- | --- | --- | --- | --- |
| 1 | 5.644 | hexanal | C6H14 | 66-25-1 | Aldehyde | 0.33±0.066 | 0.54±0.049 | 1.672 | 0.024 | 1.626 | up |
| 2 | 7.164 | (Z)-3-hexenol | C6H12O | 928-96-1 | Alcohol | 0±0 | 0.49±0.06 | 2.391 | 0.000 | Inf | up |
| 3 | 7.568 | p-xylene | C8H10 | 106-42-3 | Aromatic compound | 0.6±0.076 | 0.59±0.075 | 0.116 | 0.878 | 0.972 | insig |
| 4 | 9.392 | α-pinene | C10H16 | 80-56-8 | Monoterpene | 5.79±0.526 | 7.92±1.049 | 1.234 | 0.095 | 1.368 | insig |
| 5 | 10.185 | benzaldehyde | C10H14 | 202-860-4 | Aldehyde | 0±0 | 0.01±0.008 | 1.222 | 0.094 | Inf | insig |
| 6 | 10.48 | 3,7,7-trimethyl-1,3,5-cycloheptatriene | C10H14 | 3479-89-8 | Homoterpene | 6.06±0.575 | 8.07±1.387 | 0.959 | 0.209 | 1.331 | insig |
| 7 | 10.659 | β-pinene | C10H16 | 18172-67-3 | Monoterpene | 0.13±0.018 | 0.17±0.034 | 0.895 | 0.269 | 1.355 | insig |
| 8 | 11.006 | β-myrcene | C10H16 | 123-35-3 | Monoterpene | 1.49±0.217 | 1.63±0.222 | 0.290 | 0.657 | 1.094 | insig |
| 9 | 11.29 | 2-carene | C10H16 | 4497-92-1 | Monoterpene | 36.83±3.126 | 44.71±5.727 | 0.822 | 0.250 | 1.214 | insig |
| 10 | 11.475 | α-phellandrene | C10H16 | 99-83-2 | Monoterpene | 4.92±0.407 | 7.47±1.178 | 1.236 | 0.068 | 1.519 | insig |
| 11 | 11.781 | α-terpinene | C10H18O | 99-86-5 | Monoterpene | 0.92±0.098 | 1.6±0.316 | 1.195 | 0.069 | 1.737 | insig |
| 12 | 11.997 | p-cymene | C10H14 | 99-87-6 | Monoterpene | 0.77±0.082 | 0.94±0.103 | 0.880 | 0.228 | 1.214 | insig |
| 13 | 12.201 | β-phellandrene | C10H16 | 99-86-5 | Monoterpene | 84.98±7.659 | 100.77±8.916 | 0.922 | 0.198 | 1.186 | insig |
| 14 | 12.486 | benzeneacetaldehyde | C10H12O | 144164-15-8 | Aldehyde | 0.07±0.007 | 0.09±0.011 | 0.953 | 0.164 | 1.274 | insig |
| 15 | 12.594 | (Z)-β-ocimene | C10H16 | 13877-91-3 | Monoterpene | 0.93±0.121 | 1.21±0.142 | 1.009 | 0.144 | 1.310 | insig |
| 16 | 12.928 | γ-terpinene | C10H16 | 99-85-4 | Monoterpene | 0.41±0.03 | 0.57±0.077 | 1.146 | 0.083 | 1.386 | insig |
| 17 | 13.67 | terpinolene | C10H16 | 586-62-9 | Monoterpene | 0.47±0.04 | 0.71±0.113 | 1.124 | 0.076 | 1.504 | insig |
| 18 | 13.966 | linalool | C10H18O | 78-70-6 | Monoterpene | 0.24±0.028 | 0.22±0.025 | 0.459 | 0.679 | 0.932 | insig |
| 19 | 14.15 | nonanal | C9H18O | 124-19-6 | Aldehyde | 0.12±0.028 | 0.17±0.037 | 0.773 | 0.326 | 1.387 | insig |
| 20 | 16.052 | (Z)-3-hexenyl butanoate | C10H18O2 | 16491-36-4 | Ester | 0.14±0.012 | 0.19±0.026 | 1.277 | 0.085 | 1.378 | insig |
| 21 | 16.294 | dill ether | C10H16O | 70786-44-6 | Monoterpene | 0.23±0.038 | 0.29±0.055 | 0.562 | 0.445 | 1.224 | insig |
| 22 | 16.425 | methyl salicylate | C8H8O3 | 119-36-8 | Benzenoid ester | 0.22±0.042 | 0.26±0.06 | 0.251 | 0.603 | 1.174 | insig |
| 23 | 16.738 | decanal | C10H20O | 112-31-2 | Aldehyde | 0.09±0.015 | 0.1±0.013 | 0.524 | 0.574 | 1.125 | insig |
| 24 | 17.151 | p-cumenol | C9H12O | 99-89-8 | Alkylphenol | 0.22±0.016 | 0.21±0.026 | 0.171 | 0.802 | 0.964 | insig |
| 25 | 17.642 | p-cymen-7-ol | C10H14O | 536-60-7 | Monoterpenes | 0.08±0.011 | 0.14±0.021 | 1.540 | 0.036 | 1.691 | up |
| 26 | 18.089 | δ-elemene | C15H24 | 20307-84-0 | Sesquiterpene | 0.06±0.023 | 0.14±0.026 | 1.254 | 0.055 | 2.101 | insig |
| 27 | 18.604 | β-elemene | C15H24 | 515-13-9 | Sesquiterpene | 0.11±0.03 | 0.21±0.038 | 1.238 | 0.057 | 1.922 | insig |
| 28 | 21.753 | α-cedrene | C15H24 | 469-61-4 | Sesquiterpene | 0.14±0.016 | 0.13±0.009 | 0.539 | 0.505 | 0.910 | insig |
| 29 | 21.816 | (E)-β-caryophyllene | C15H24 | 87-44-5 | Sesquiterpene | 3.97±0.676 | 2.8±0.27 | 1.118 | 0.136 | 0.704 | insig |
| 30 | 21.935 | γ-elemene | C15H24 | 29873-99-2 | Sesquiterpene | 0.09±0.013 | 0.08±0.01 | 0.442 | 0.511 | 0.876 | insig |
| 31 | 22.25 | 6,9-guaiadiene | C15H24 |  | Sesquiterpene | 0.19±0.04 | 0.17±0.018 | 0.119 | 0.724 | 0.915 | insig |
| 32 | 22.577 | α-caryophyllene | C15H24 | 6753-98-6 | Sesquiterpene | 0.75±0.123 | 0.52±0.049 | 1.132 | 0.123 | 0.703 | insig |
| 33 | 24.503 | trans-farnesol | C15H26O | 106-28-5 | Sesquiterpene | 0.04±0.009 | 0.03±0.008 | 0.091 | 0.863 | 0.940 | insig |
| 34 | 24.877 | TMTT | C16H26 | 62235-06-7 | Terpenoid | 4.31±0.953 | 3.26±0.944 | 0.481 | 0.444 | 0.756 | insig |
| 35 | 25.11 | diethyl phthalate | C12H14O4 | 84-66-2 | Ester | 0.62±0.115 | 0.54±0.106 | 0.144 | 0.610 | 0.869 | insig |

Note: Following discriminant analysis of orthogonal partial least squares with threshold of *P*-value < 0.05, Fold_change ≥ 1.5 and variable importance in projection (VIP) ≥ 1. Show the mean ± SE from 9 replicates.

**Table S4.** qRT-PCR primers of 8 selected genes in tomato.

| Gene Name | Forward primers (5’ to 3’) | Reverse primers (5’ to 3’) |
| --- | --- | --- |
| *LOC101245524* | 5’- ggcctcgtattggttgtgtt -3’ | 5’- gaggggatagggccatacat -3’ |
| *LOC101250825* | 5’- cggaaaaagatgggtgaaaa -3’ | 5’- ttcccttcgactggaacttg -3’ |
| *LOC101255217* | 5’- aacaagccacctgaaccatc -3’ | 5’- ccctttgcagaggagagcta -3’ |
| *LOC101256653* | 5’- aacaagccacctgaaccatc -3’ | 5’- cagcaatggctgactttgaa -3’ |
| *LOC101256903* | 5’- gcttggcaagaacctgctac -3’ | 5’- atgaccaccatgcaactgaa -3’ |
| *LOC101262338* | 5’- gcatgaagctgtgaaggaaa -3’ | 5’- gcaccccctctaacaaatga -3’ |
| *LOC101265748* | 5’- atcacgaagccgaagaagaa -3’ | 5’- agggccacaacagaatcttg -3’ |
| *TUB* | 5’- ttggttttgcaccactgacttc -3’ | 5’- aagctctggcactgtcaaagc -3’ |

**Table S5.** Primers used for VIGS.

| Genes | Accession number | Forward primers | Reverse primers |
| --- | --- | --- | --- |
| *TRV2-IQM1* | *LOC101245524* | 5’- gtgagtaaggttaccgaattcatgactgttcgatcgaatagctttaa -3’ | 5’- cgtgagctcggtaccggatcccttatacactttctgaagcttaacagca -3’ |
| *TRV2-GFP* | *HM151400.1* | 5’- gtgagtaaggttaccgaattcATGAGTAAAGGAGAAGAACT -3’ | 5’- cgtgagctcggtaccggatccTTTGTATAGTTCATCCATGCC -3’ |

**Table S6.** Primers used for DNA constructs.

| Genes | Accession number | Forward primers | Reverse primers |
| --- | --- | --- | --- |
| *VC-LOC101245524* | *LOC101245524* | 5’- cagtGGTCTCaagtgcgtggagaaaacagaatcgagttttagagc -3’ | 5’- cagtGGTCTCaaaacccgcgatacagcagtttccgtg -3’ |
| *Test-LOC101245524* | *LOC101245524* | 5’- GATATGTTGTTGTTGTAATGTTC -3’ | 5’- TGTCGTGGATCAATCTAGAC -3’ |

Note: homologous recombination was used for constructing the pHSbdcas9i vectors. The restriction sites are BsaI and Eco31I.

**Table S7.** Life table parameters of of *Phthorimaea absoluta* on wild type and IQM1 mutant tomato plants.

| Parameters | WT | MT2-1 | *t* | *df* | *P* |
| --- | --- | --- | --- | --- | --- |
| Egg duration (d) | 4.59±0.058 | 4.73±0.895 | 1.311 | 98 | 0.193 |
| Egg survival (%) | 96.00±2.211 | 94.00±3.399 | 0.493 | 18 | 0.628 |
| Larval duration (d) | 10.45±0.172 | 10.65±0.199 | 0.760 | 98 | 0.449 |
| Larval survival (%) | 74.00±3.055b | 82.00±2.000a | 2.138 | 18 | **0.046** |
| Female pupae duration (d) | 6.81±0.096 | 6.66±0.102 | 1.070 | 98 | 0.287 |
| Male pupae duration (d) | 7.21±0.139 | 6.99±0.095 | 1.303 | 98 | 0.195 |
| Pupal survival (%) | 95.00±3.073 | 91.00±4.069 | 0.784 | 18 | 0.443 |
| Sex ratio (female : male) | 51.00±2.769 | 48.00±2.494 | 0.805 | 18 | 0.431 |
| Female longevity (d) | 10.10±0.672 | 10.90±0.636 | 0.864 | 38 | 0.393 |
| Male Longevity (d) | 10.95±0.618 | 10.40±0.617 | 0.63 | 38 | 0.533 |
| Total fecundity | 139.87±9.654 | 129.13±10.124 | 0.553 | 28 | 0.585 |

Note: WT: wild type tomato plants; MT2-1: IQM1 mutant tomato plants. Life table parameters between WT and MT2-1 were compared by *t*-test (*P* < 0.05).
